# Nonreciprocal Negative Refraction Enabled by Photonic Time Crystals

**DOI:** 10.1021/acs.nanolett.5c06214

**Published:** 2026-01-23

**Authors:** Mohammad R. Tavakol, Wenshan Cai

**Affiliations:** † School of Electrical and Computer Engineering, 1372 Georgia Institute of Technology, Atlanta, Georgia 30332, United States; ‡ School of Materials Science and Engineering, 1372 Georgia Institute of Technology, Atlanta, Georgia 30332, United States

**Keywords:** Metamaterials, negative refraction, photonic time crystals, nonreciprocity, time modulation

## Abstract

We propose and theoretically demonstrate nonreciprocal negative refraction enabled by time-varying photonic structures. By engineering temporal modulations at the interfaces of hyperbolic media, we achieve isolation between forward and backward beams while preserving the hallmark property of negative refraction. Two complementary approaches are developed: in the optical regime, a multilayer AZO/ZnO hyperbolic slab is sandwiched between permittivity-modulated dielectric layers (3D time crystals); in the microwave regime, a wire medium is sandwiched between time-modulated resistive metasurfaces (2D time crystals). Both designs exploit Floquet harmonic expansions and are validated with a customized harmonic-balance finite-element solver. We report isolation exceeding 46 dB in the optical device and 11 dB in the microwave counterpart. This work introduces a general framework for nonreciprocal negative refraction across frequency regimes, expanding the design space of time-varying metasurfaces and photonic time crystals.

## Graphical abstract

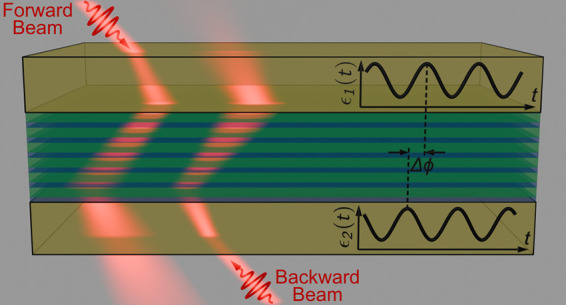

The concept of negative refraction has long fascinated the photonics community because of its ability to fundamentally alter the way electromagnetic waves propagate. Following Veselago’s theoretical predictions, experimental realizations of negative-index metamaterials enabled unprecedented control over light propagation and established new regimes of optics.^1–3^ Negative refraction enables a range of unusual wave phenomena, including reversed Cherenkov radiation, negative Goos–Hänchen shifts, backward-wave propagation, and negative radiation pressure.^4^ Among the various approaches to realizing negative refraction, *hyperbolic media* stand out because of their highly anisotropic dispersion relations, which support both propagating and evanescent modes and give rise to backward-wave refraction.^5^ These properties have been exploited in multilayer plasmonic and semiconductor systems, including transparent conducting oxide multilayers such as aluminum-doped zinc oxide (AZO)/ZnO stacks, which offer tunability and compatibility with nanophotonic platforms.^6^

Despite such progress, conventional negative refraction remains fundamentally *reciprocal*. Lorentz reciprocity dictates that under linear and time-invariant conditions, electromagnetic systems respond identically to forward and backward excitation.^7^ Thus, a beam incident from the forward direction experiences the same refraction angle and transmission efficiency as one entering from the backward direction, which prevents any inherent directionality in the response.

The traditional route to breaking reciprocity is through magneto-optical effects, in which time-reversal symmetry is broken by applying an external magnetic bias.^8^ Magneto-optical devices are widely used in microwave engineering and optical communication, yet they suffer from severe drawbacks: they are bulky, require strong magnetic fields, and are challenging to integrate on-chip. This has fueled intense efforts to achieve magnetic-free nonreciprocity.^9^

Among various approaches, *temporal modulation* has emerged as a particularly promising strategy.^10–14^ Temporally varying permittivity, permeability, or conductivity breaks time-reversal symmetry directly and couples the incident frequency to sidebands at harmonics of the modulation frequency.^15^ Unlike static anisotropy or geometric asymmetry, which preserve reciprocity, temporal modulation induces frequency conversion and directional asymmetry, since forward and backward waves interact with the modulation phase differently. Related transmissive time-modulated media and space–time Bragg gratings have been shown to exhibit robust optical nonreciprocity and compact nonreciprocal responses.^16–18^ Theoretically, even uniform time modulation in bianisotropic systems can produce nonreciprocity under appropriate coupling conditions, reinforcing the generality of temporal-symmetry breaking.^19^

The concept of *photonic time crystals (PTCs)*—systems with periodic modulation in time—naturally extends photonic crystals into the temporal domain. Just as spatial periodicity produces photonic bandgaps, temporal periodicity creates frequency bandgaps and exotic dispersion properties.^20^ Theoretical work has predicted amplification, frequency conversion, and temporal Bragg scattering in such systems.^21,22^ Recent experiments have confirmed aspects of these predictions, showing the feasibility of dynamic index modulation on subcycle time scales.^23^ Diverse mechanisms have been explored: carrier depletion in semiconductors,^24^ photon acceleration in transparent conducting oxides,^25^ and time-varying surface impedances in microwaves.^26^ For example, varactor-loaded or resistive patch arrays have been used to implement time-varying admittances, producing strong nonreciprocal responses in compact microwave devices.^27,28^ Theoretical frameworks based on generalized transfer matrices,^29^ modal methods,^30,31^ and transmission-line circuit models^26,32,33^ have unified the treatment of such temporal and spatiotemporal systems, further broadening their applicability. Additionally, parallel advances in spatiotemporal metasurfaces further highlight the utility of temporal modulation.^34–36^ These platforms combine spatial structuring with time modulation to realize asymmetric transmission, and frequency conversion.^30,32,37–39^

Despite these advances, *nonreciprocal negative refraction* has not been achieved. Existing works fall into two distinct categories: negative refraction in static hyperbolic or negative-index systems (reciprocal),^1,6^ and nonreciprocity in temporally modulated systems (without negative refraction).^15,28,40^ Achieving directional control in systems that refract light negatively would expand the functionality of negative-index and hyperbolic platforms, enabling asymmetric beam steering, isolating components, and robust signal-routing capabilities that static negative-refraction systems fundamentally lack. Combining the two requires careful integration of hyperbolic dispersion with temporal modulation to maintain negative refraction while breaking reciprocity.

In this work, we present the demonstration of nonreciprocal negative refraction enabled by time crystals. Our approach leverages temporal modulation as a symmetry-breaking tool that enables nonreciprocal control of negatively refracted beams. To implement this concept, we sandwich a hyperbolic medium between two temporally modulated interfaces driven with a quadrature-phase offset (90°). This configuration ensures that forward and backward beams encounter distinct temporal states of the system. As a result, both directions undergo negative refraction, but their transmission amplitudes differ strongly, yielding isolation. Together, these features unite the physics of hyperbolic dispersion with symmetry breaking from time modulation, establishing a general framework for nonreciprocal negative refraction.

We exemplify the concept in two complementary frequency regimes. In the optical regime, we design a multilayer AZO/ZnO hyperbolic stack bounded by permittivity-modulated dielectric slabs, which act as *3D time crystals*. This system leverages reactive modulation, where energy is stored and redistributed among harmonics. Rigorous theory and simulations confirm isolation exceeding 46 dB while maintaining negative refraction. In the microwave regime, we use a wire medium bounded by resistive metasurfaces whose sheet conductances are temporally modulated with a quadrature-phase offset. This represents a *2D time crystal* that leverages absorptive modulation, where direction-dependent dissipation enforces nonreciprocity. The achieved isolation is ∼ 11 dB, smaller than in the optical case, but the microwave implementation permits much stronger temporal modulation, which is easier to realize in conductance-modulated metasurfaces than in optically modulated dielectrics.

Together, these two platforms exhibit the universality of the framework. By integrating hyperbolic dispersion with time-crystal interfaces, we achieve a previously unrealized form of nonreciprocal negative refraction. This approach opens pathways toward magnet-free isolators, asymmetric beam routing devices, and integrated nonreciprocal components, while also extending the physics of photonic time crystals into new regimes.

We begin by outlining the general concept of time-crystal-enabled nonreciprocal negative refraction. Figure 1 illustrates the core idea. A TM-polarized plane wave at frequency ω_0_ impinges on a hyperbolic slab that is bounded on both sides by time-varying interfaces. Because of temporal modulation, the incident frequency couples to sidebands ω_*m*_ = ω_0_ + *m*Ω, exciting a spectrum of harmonics in both reflection and transmission. These two interfaces with distinct temporal phases will be employed to break the reciprocity of negative refraction through the hyperbolic region.

**Figure 1:**
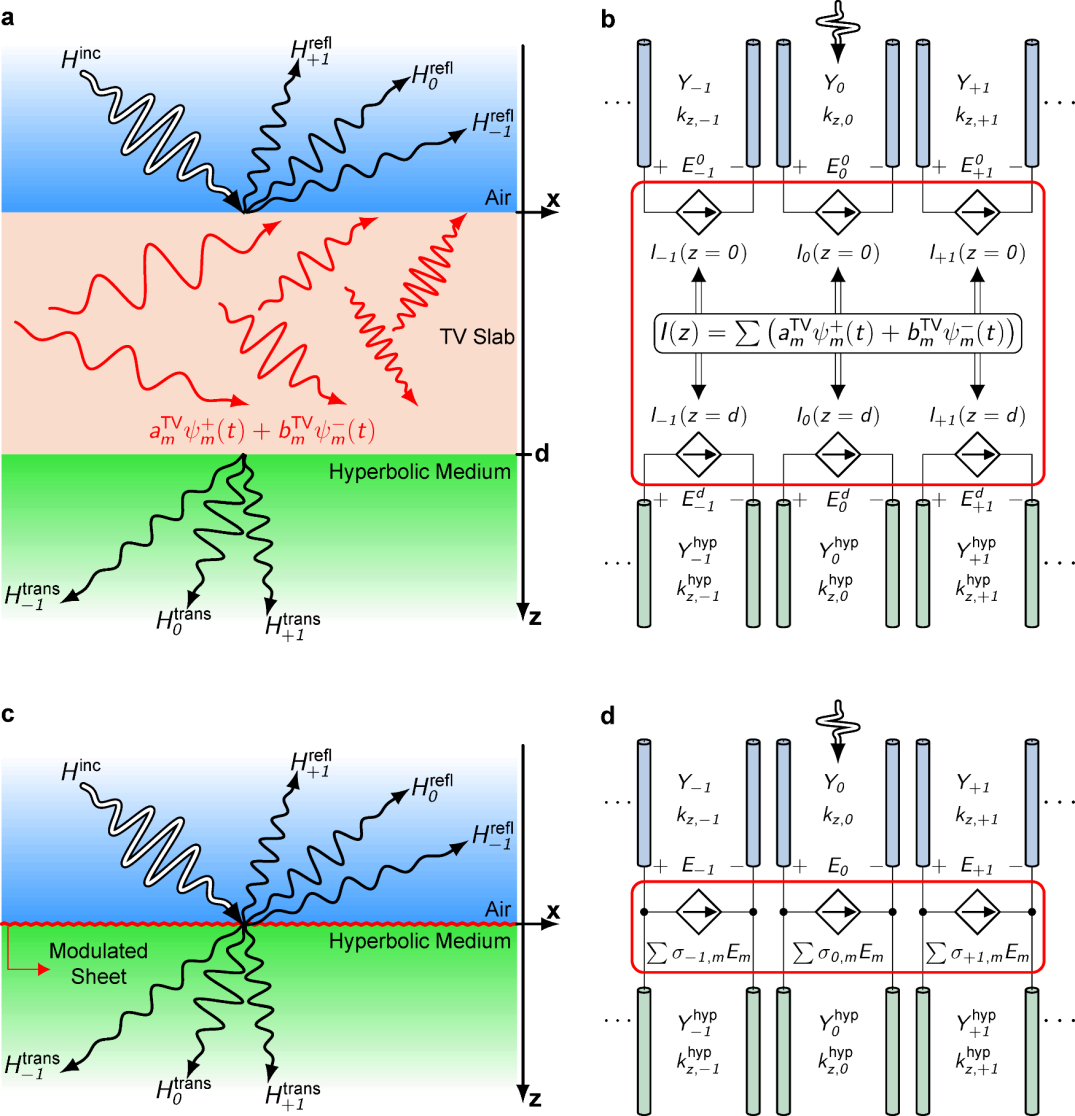
Modeling time-varying interfaces through negative refraction. (a) Schematic of a multilayer structure where a TM-polarized wave impinges from air (blue) onto a time-varying (TV) dielectric slab of thickness *d* (red) adjacent to a hyperbolic medium (green). The incident beam (thick white arrow) excites reflected and transmitted harmonics (black arrows). Inside the TV slab, the field is a superposition of forward and backward multiharmonic modes, ψ_*m*_^+^(*t*) and ψ_*m*_^–^(*t*), with amplitudes *a*_*m*_^TV^ and *b*_*m*_^TV^. Different harmonics of these modes are shown by the red arrows. Black and red wavy arrows with varying oscillation periods denote distinct harmonics. The structure is homogeneous along the *y*-axis. (b) Equivalent circuit model of (a). Each harmonic in the air and hyperbolic regions is represented by a transmission line with propagation constant *k*_*z*,*m*_ and wave admittance *Y*_*m*_. The incident excitation couples into the zeroth-order line of the air region. The TV slab is modeled (red box) as a linear combination of forward and backward harmonics, with voltage-controlled current sources mediating interharmonic coupling. Three dots before and after the lines indicate that only a subset of the infinite harmonic spectrum is shown. (c) Schematic of a configuration with a time-modulated conductive sheet (red line) at the air–hyperbolic interface. Unlike the TV slab in (a), the sheet generates multiharmonic surface currents that couple the incident excitation to reflected and transmitted harmonics. Similar to (a), the structure is homogeneous along the *y*-axis. (d) Equivalent circuit model of (c). Transmission lines again represent harmonics in the air and hyperbolic regions. The time-modulated sheet is modeled (red box) by shunt admittances realized through voltage-controlled current sources, which directly couple harmonics across the interface.

For a 3D time crystal region as the interface, the modulation of permittivity in the dielectric slab regions is described by $\epsilon_{l}\left(t\right)=\epsilon_{r,0}+\Delta \epsilon \;\cos\left(\Omega t+\phi_{l}\right)$ (*l* = 1, 2), representing the top and the bottom slabs. As shown schematically in Figure 1(a), the incident TM wave excites forward- and backward-propagating multiharmonic eigenmodes inside the time-varying (TV) slab. These are denoted ψ_*m*_^+^(*t*) = ψ_*m*_^TV^(*t*)*e* ^–*jk*_*z*,*m*_^TV^ *z*^ and ψ_*m*_^–^(*t*) = ψ_*m*_^TV^(*t*)*e* ^+*jk*_*z*,*m*_^TV^ *z*^, with amplitudes *a*_*m*_^TV^ and *b*_*m*_^TV^, respectively. Their superposition can be written as ∑_*m*_(*a*_*m*_^TV^ψ_*m*_^+^(*t*) + *b*_*m*_^TV^ψ_*m*_^–^(*t*)). These modes mediate coupling into reflected and transmitted harmonics in the air and hyperbolic regions, indicated by the black wavy arrows of different periods. In this way, the TV slab generates multiple frequency channels, each corresponding to a Floquet harmonic.

The equivalent circuit model in Figure 1(b) provides a compact representation. Each harmonic *m* in the air region is modeled by a transmission line with propagation constant $k_{z,m}=\sqrt{{\left(\omega_{m}/c\right)}^{2}-k_{x,\mathrm{inc}}^{2}}$ , where ω_*m*_ = ω_0_ + *m*Ω, and characteristic admittance *Y*_*m*_ = (*k*_*m*_/*k*_*z*,*m*_)­(1/η) with *k*_*m*_ = ω_*m*_/*c* and η the free-space impedance. Similarly, each harmonic in the hyperbolic region is represented by a line with $k_{z,m}^{\mathrm{hyp}}=\sqrt{\epsilon_{t}\left(\omega_{m}^{2}/c^{2}-k_{x,\mathrm{inc}}^{2}/\epsilon_{z}\right)}$ and *Y*_*m*_^hyp^ = (*k*_*m*_/*k*_*z*,*m*_^hyp^)­(1/η). The red box in Figure 1(b) represents the TV slab, which is modeled as a set of voltage-controlled current sources at *z* = 0 and *z* = *d*. Each source is a linear combination of the harmonic electric field amplitudes at the slab boundaries, *E*_*m*_^0^ and *E*_*m*_^*d*^, and is determined by enforcing field boundary conditions. In this picture, the transmission lines represent the time diffraction orders, while the controlled sources capture harmonic mixing induced by modulation.

For a 2D time crystal boundary as the interface, as shown in Figure 1(c), the time-varying interfaces are resistive or conductive sheets whose conductance is modulated as $\sigma_{l}\left(t\right)=\sigma_{0}+\Delta \sigma \;\cos\left(\Omega t+\phi_{l}\right)$ (*l* = 1, 2). Here, the mechanism is similar but simpler. The sheet generates surface currents that directly couple the incident excitation to reflected and transmitted harmonics. In the circuit representation (Figure 1(d)), this appears as shunt admittances (red box) that connect harmonics in the air and hyperbolic regions. The coupling currents are determined by convolution of the Toeplitz matrix associated with $\sigma_{l}\left(t\right)$ with the boundary field amplitudes *E*_*m*_. Thus, while the slab case involves distributed volumetric modulation, the sheet case involves surface currents that directly enforce asymmetric coupling across the interface.

The thick white arrow in Figures 1(a) and 1(c) denotes the oblique incident wave at frequency ω_0_, while the thinner wavy arrows denote reflected and transmitted harmonics. In both the slab and sheet configurations, the key principle is that time modulation produces a ladder of Floquet harmonics, some propagating and others evanescent. With a π/2 phase offset between the two modulated boundaries, these harmonics interfere constructively in one direction (forward) and destructively in the other (backward). This interference results in strong direction-dependent transmission while retaining the negative refraction associated with the hyperbolic core.

Taken together, Figure 1 and the above formulations show how time-varying boundaries on a hyperbolic medium transform reciprocal negative refraction into a nonreciprocal effect. In the optical design, the modulated slabs act as 3D photonic time crystals where volumetric modulation excites multiharmonic eigenmodes. In the microwave design, the modulated sheets act as 2D photonic time crystals where surface currents enforce asymmetric coupling. Both approaches implement the same principle: interference between Floquet harmonics controlled by quadrature modulation, yielding nonreciprocal negative refraction. The following subsections illustrate these principles concretely in the optical (Figure 2) and microwave (Figure 4) regimes. While the discussion here focuses on TM polarization, the proposed framework can be extended to TE-polarized excitation with modified admittance definitions and eigenmode dispersion relations, as detailed in the Supporting Information.

**Figure 2:**
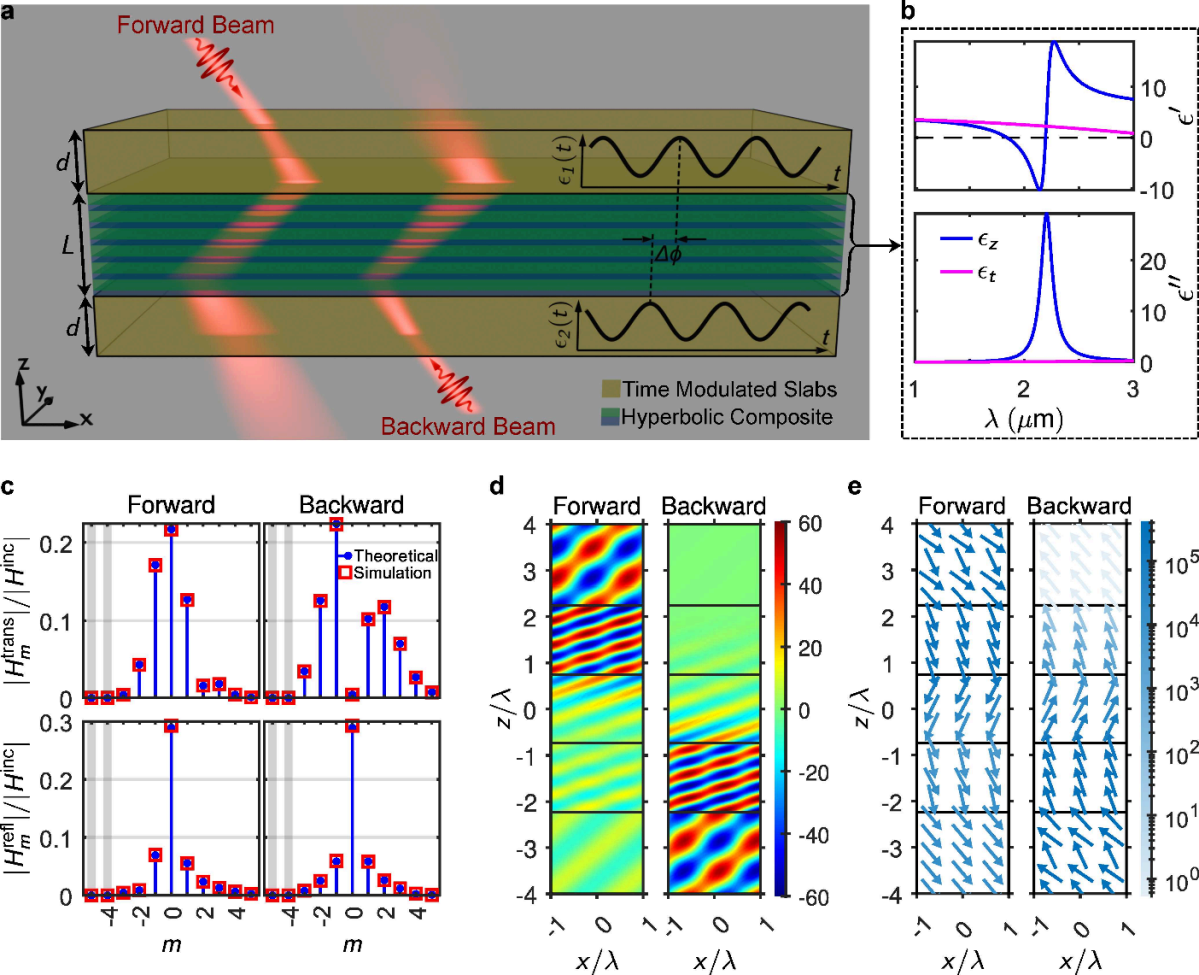
Working principle and typical response of the proposed nonreciprocal negative refraction in the optical range. (a) A schematic of the structure composed of an alternating layer stack of thin plasmonic and dielectric films (AZO/ZnO), sandwiched between two dielectric slabs with modulated permittivity. Besides the subwavelength features of the hyperbolic layer, the system is mainly characterized by the hyperbolic slab thickness, *L* = 2.8 μm, and the incident angle, θ_inc_ = 40°. The permittivities of the two modulated slabs are varied with the same frequency and modulation depth but have different phases, i.e., ϕ_1_ and ϕ_2_. The forward and backward oblique beams exhibit significantly different transmissions, resulting in nonreciprocal behavior. The red light beams schematically depict diffraction (due to harmonic modulations) and refraction through the structure. (b) The real and imaginary parts of the effective perpendicular and transverse permittivities of the thin film stack, used as the hyperbolic medium to realize negative refraction. The periodicity of the thin films along the structure is subwavelength, with a metal filling factor of *f* = *t*_*m*_/(*t*_*m*_ + *t*_*d*_) = 0.3, where *t*_*m*_ and *t*_*d*_ are the thicknesses of the metal and dielectric layers, respectively. (c) Forward and backward transmission and reflection field amplitudes, obtained through simulation and analytical calculation, versus harmonic order, *m*, with each frequency harmonic ω_*m*_ being ω_0_ + *m*Ω. (d) The magnetic field profile (*Hy*) when the structure is illuminated from the top (forward) and bottom (backward). Both surface plots use the same linear scale, shown via the colorbar on the right. (e) Power vector fields corresponding to the field profile in (d). The vector fields are normalized, and the arrow colors indicate power amplitude. Both vector field plots share the same linear scale, represented by the colorbar on the right.

We first consider the optical implementation, which employs a multilayer AZO/ZnO hyperbolic slab sandwiched between two permittivity-modulated dielectric layers. AZO is a transparent conducting oxide with metallic response in the near-IR, while ZnO serves as the dielectric partner. When alternated in thin layers, the composite exhibits hyperbolic dispersion, enabling negative refraction for TM waves.

The structure is shown schematically in Figure 2(a). The hyperbolic dispersion relation, calculated via effective medium theory (see ), is plotted in Figure 2(b), revealing Type-II hyperbolicity near λ = 1.9 μm. These multilayers have been widely studied as tunable hyperbolic metamaterials,^1^ and their integration with time-modulated interfaces marks an unexplored frontier in coupling hyperbolic dispersion and temporal modulation across distinct regions.

Figures 2(c)–(e) present the central results. Under forward incidence, the zeroth-order transmission dominates, and the beam undergoes negative refraction with moderate insertion loss. Under backward incidence, by contrast, the zeroth-order transmission is strongly suppressed. Figure 2(c) plots the harmonic spectra, where forward illumination yields significant zeroth-order transmission, while backward illumination is nearly extinguished. As observed in Figure 2(c), backward illumination leads to a pronounced redistribution of energy among the Floquet harmonics as a result of the temporal modulation. Due to modulation-induced coupling between neighboring sidebands, the backward-propagating *m* = 0 harmonic undergoes destructive interference and is therefore strongly suppressed in transmission, whereas the *m* = – 1 harmonic experiences constructive interference and consequently emerges as the dominant transmitted component. Field maps of *H*_*y*_ in Figure 2(d) show a clear negatively refracted beam for forward incidence, while the backward case produces only weak scattered fields. Poynting vector distributions in Figure 2(e) confirm the effect, revealing direction-dependent energy flow through the structure.

We quantify the asymmetry using the isolation metric

$$I=10\;\log_{10}\left(\frac{T_{f}}{T_{b}}\right)$$ 1

where *T*_*f*_ and *T*_*b*_ are the forward and backward zeroth-order power transmission coefficients. At the operating wavelength, we obtain *I* ≈ 46.4 dB, establishing strong nonreciprocity while preserving negative refraction.

A practical device must balance isolation with forward insertion loss. To capture this trade-off, we define a figure of merit (FoM) as

$$\mathrm{FoM}=\alpha_{1}I+\alpha_{2}10\;\log_{10}\left(T_{f}\right),\alpha_{1}+\alpha_{2}=1$$ 2

where we assume α_1_ = α_2_ = 0.5 in our analysis. Figure 3(a) shows a two-dimensional map of the figure of merit (FoM) as a function of normalized modulation frequency Ω/ω_0_ and hyperbolic thickness *L*. Figure 3(b) presents the FoM and the zeroth-order transmission as functions of wavelength detuning, illustrating how performance varies around the design point. Optimal performance is achieved when the modulation frequency is tuned near Ω/ω_0_ = 0.1 and *L* ≈ 1.5λ, consistent with design constraints in our configuration.

**Figure 3:**
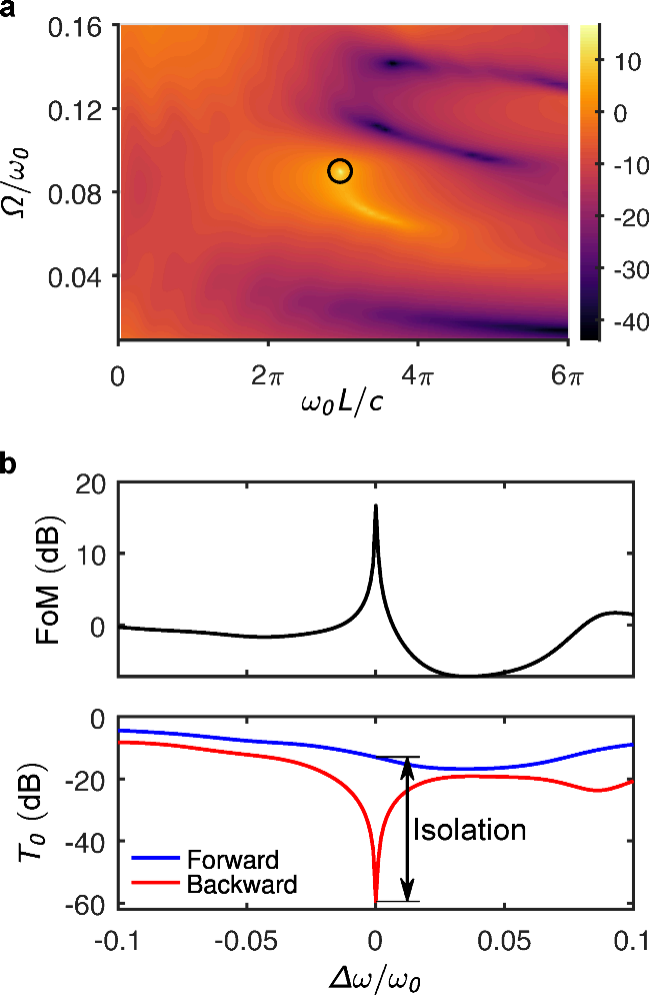
Performance analysis of the optical design. (a) Defined figure of merit (FoM) as a function of the hyperbolic region thickness (normalized to free-space wavelength by multiplication with *k*_0_ = ω_0_/*c*) and the modulation frequency (normalized to the optical frequency ω_0_). The FoM (color scale) is expressed in dB. The black circle marks the configuration with the maximum FoM. (b) FoM and zeroth-order transmission as a function of frequency detuning, both in dB scale. The isolation is quantified by *I* = 46.4 dB.

These results highlight the ability of permittivity-modulated slabs to realize strong nonreciprocity at optical frequencies, leveraging reactive modulation to couple harmonics without requiring extreme material parameters, i.e., smaller modulation depths Δϵ/ϵ_*r*,0_ = 0.1. The integration of AZO/ZnO hyperbolic multilayers with dynamic dielectric interfaces represents a new paradigm for tunable optical isolators and beam shapers. We note that, unlike the idealized temporal coupled-mode theory (TCMT) scenario of ref 40, our two time-varying slabs are optically thick $\left(\sqrt{\epsilon_{r,0}}d=3\lambda \right)$ and weakly coupled through a dispersive hyperbolic core. The resulting multimode interaction adds internal propagation phases to the effective modulation channels, so that the optimal modulation phase offset for maximum isolation is slightly shifted from the nominal quadrature value. In our design, the FoM peaks at ϕ_2_ – ϕ_1_ ≈ π/2 + π/50, which we attribute to these higher-order coupling and finite-thickness effects.

To demonstrate the generality of our framework for achieving time-crystal–enabled nonreciprocal negative refraction, we translate the concept into the microwave regime using conductance-modulated metasurfaces. Here the hyperbolic medium is realized as a wire metamaterial, which is well-known to support hyperbolic dispersion at GHz frequencies.^41^ The interfaces are implemented as resistive sheets whose conductances vary sinusoidally in time, driven by a local oscillator split by a Wilkinson divider and delayed by a 90° phase shifter (Figure 4(a)). This approach closely follows prior designs of time-varying metasurfaces.^28,42^

**Figure 4:**
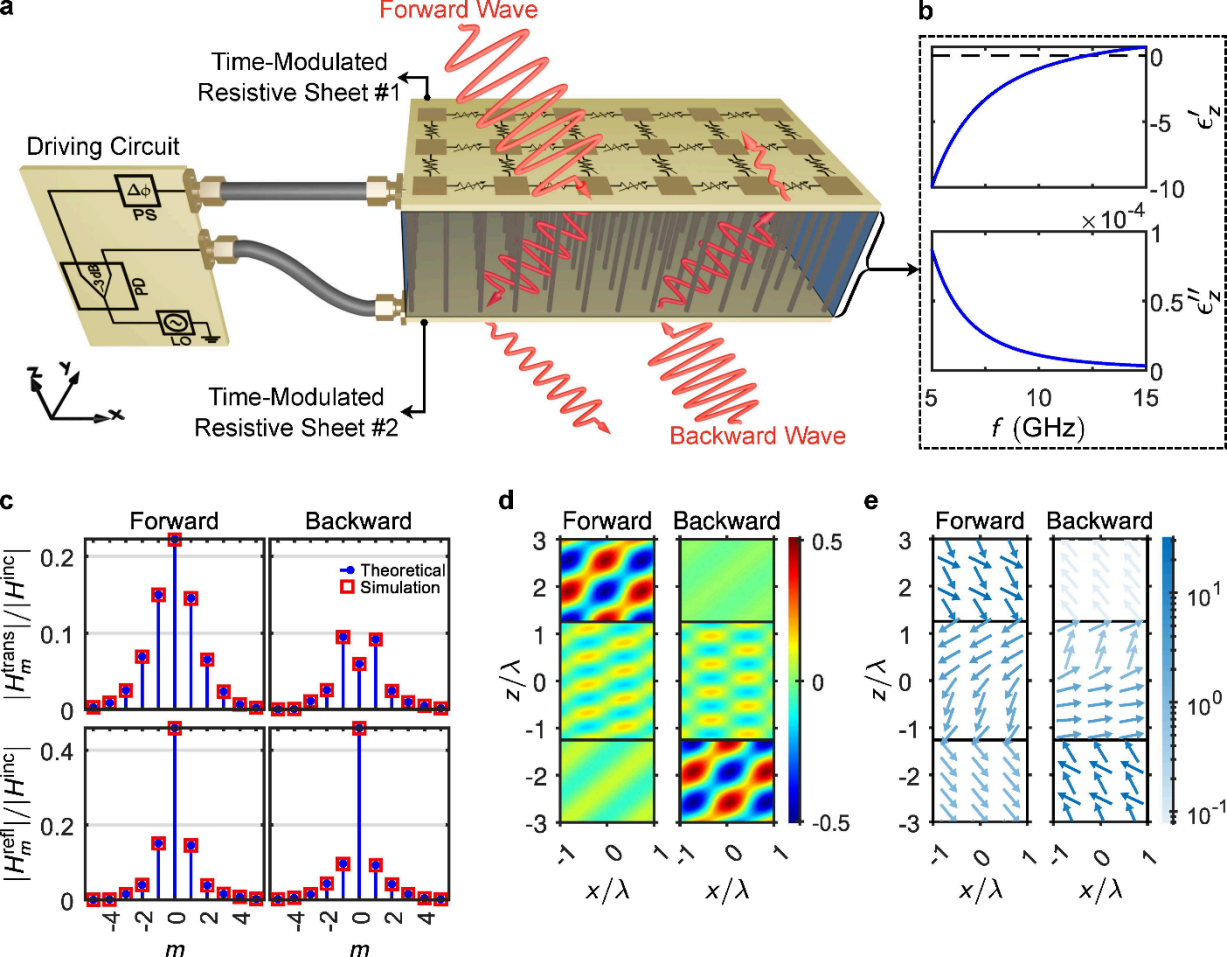
Conceptual illustration and representative response of the proposed approach for achieving nonreciprocal negative refraction in the microwave domain. (a) Schematic of the structure made by a wire medium sandwiched by two sheets whose conductance is modulated. Apart from the subwavelength structural parameters of the hyperbolic layer, this system is defined by only two geometrical parameters: hyperbolic medium thickness, *L*, and the incident angle θ_inc_ = 40°. The driving circuit, consisting of a source with a modulation frequency of Ω, a power divider (PD), and a phase shifter (PS), provides temporal modulations with different phases. The forward and backward oblique beams exhibit very different transmissions, resulting in nonreciprocity. The red beams (wavy arrows) qualitatively indicate diffraction induced by temporal harmonics and refraction through the structure. (b) Extracted real and imaginary components of the effective transverse permittivity of the wire medium, serving as the hyperbolic layer to enable negative refraction. The lattice constant is *a* = 4 mm along both x and y, with wire radius *r* = 0.2 mm. (c) Simulated and analytically computed amplitudes of the transmitted and reflected fields for both illumination directions, plotted against harmonic index *m*, corresponding to frequency components ω_*m*_ = ω_0_ + *m*Ω. (d) Spatial profiles of the magnetic field component *H*_*y*_ for forward (top) and backward (bottom) illumination scenarios. Both plots share a common linear color scale shown on the right. (e) Time-averaged Poynting vector fields corresponding to the field maps in panel (d), where arrow direction and color denote energy flow direction and magnitude, respectively. The same logarithmic color scale is used for both cases and displayed on the right.

Figure 4(b) shows the effective permittivity of the wire medium, confirming hyperbolicity at 10 GHz (derivation in Supporting Information). Figures 4(c)–(e) present the spectral and spatial results. Harmonic spectra in Figure 4(c) reveal ∼11.4 dB isolation between forward and backward zeroth-order transmission. Field maps (Figure 4(d)) show negative refraction in both directions but with much weaker amplitude in the backward case. Poynting vector maps (Figure 4(e)) reinforce this conclusion, visualizing direction-dependent power flow.

The performance difference compared to the optical device is instructive. While the optical implementation achieved >40 dB isolation, the microwave version provides ∼11 dB. However, conductance modulation can reach depths as large as Δσ/σ_0_ = 0.9, far exceeding typical permittivity modulation depths in optics. This ease of achieving strong modulation makes microwave implementations highly practical. Moreover, the sheet-based architecture is significantly simpler than multilayer stacks, lowering fabrication barriers.

Figures 5(a) and 5(b) plot the FoM (eq 2) across design parameters, identifying operating regimes that maximize isolation while retaining acceptable insertion loss. These maps reveal that even modest detunings can strongly affect performance, emphasizing the importance of careful phase control in the modulation signals.

**Figure 5:**
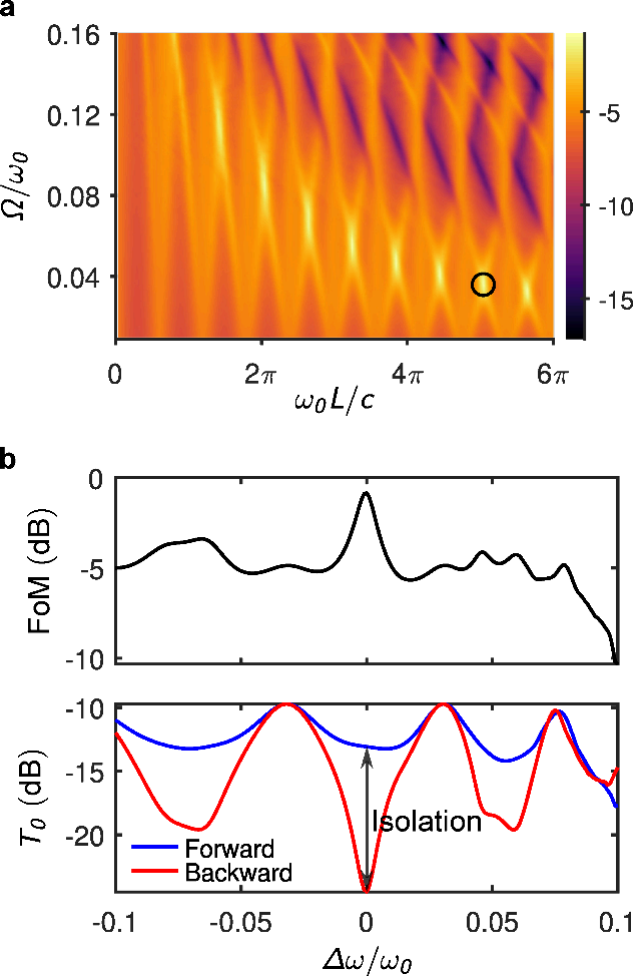
Performance analysis of the microwave design. (a) Figure of merit (FoM) as a function of the hyperbolic region thickness, normalized through *k*_0_ = ω_0_/*c*, and the modulation frequency, normalized to the wave frequency ω_0_. The FoM, shown on a dB scale, highlights the parameter space leading to the optimal performance. (b) Frequency detuning dependence of the FoM and zeroth-order transmission, both in dB. The design achieves an isolation of *I* = 11.4 dB, arising from the two time-modulated sheets.

It is worth studying the microwave device’s mechanism and performance in more detail. As mentioned, since the conductive sheets are modulated, the multiharmonic generation is dependent on the absorptions of the sheets. In fact, when the sheets’ conductivities vary with the modulation frequency, Ω, ohmic losses at different harmonics contribute to one another, collectively producing nonreciprocity at the operating (zeroth-order) frequency, ω_0_. To visualize how the total absorption behaves with frequency, we plotted it in the Supporting Information.

We have theoretically demonstrated a unified framework for achieving nonreciprocal negative refraction by embedding hyperbolic media between temporally modulated interfaces. The optical implementation, based on permittivity-modulated slabs, leverages reactive modulation to redistribute energy among harmonics and achieves isolation beyond 46 dB. The microwave implementation, based on conductance-modulated metasurfaces, employs absorptive modulation that enforces direction-dependent dissipation, yielding isolation of ∼ 11 dB.

Together, these results establish the universality of time-crystal–enabled nonreciprocal negative refraction across different frequency regimes and modulation mechanisms. This work demonstrates that temporal modulation can break reciprocity while preserving and shaping negative refraction, thereby expanding the fundamental landscape of photonic time crystals and revealing new possibilities for dynamic control of wave propagation.

## Methods

### Theoretical Analysis

Our analysis relies on a Floquet-harmonic expansion of the TM-polarized electromagnetic fields, i.e., nonzero components are *E*_*x*_, *E*_*z*_, and *H*_*y*_ (*H*_*x*_ = *H*_*z*_ = *E*_*y*_ = 0), in configurations with time-varying domains and/or boundaries. This approach is standard for periodically modulated systems, where the modulated areas or elements with frequency of Ω enforce quasi-energy conservation and produce sidebands at ω_*m*_ = ω_0_ + *m*Ω.^21,42^

In a time-varying homogeneous region or slab under a TM illumination with the transverse wavevector of *k*_*x*,inc_, the magnetic field takes the multiharmonic form of

$$\begin{array}{rll} H_{y}^{\mathrm{TV}}\left(x,z;t\right) & =\underset{m}{\sum }H_{y,m}\left(x,z\right)e^{j\omega_{m}t} & \\ & =e^{-jk_{x,\mathrm{inc}}x}\underset{m}{\sum }\left(a_{m}^{\mathrm{TV}}e^{-jk_{z,m}^{\mathrm{TV}}z}+b_{m}^{\mathrm{TV}}e^{+jk_{z,m}^{\mathrm{TV}}z}\right)\psi_{m}^{\mathrm{TV}}\left(t\right) \end{array}$$ 3

where *a*_*m*_^TV^ and *b*_*m*_^TV^ are forward and backward modal coefficients, *k*_*z*,*m*_^TV^ are the longitudinal propagation constants, and ψ_*m*_^TV^(*t*) are the multiharmonic eigenmode associated with *k*_*z*,*m*_^TV^. It is worth remarking that ψ_*m*_^±^(*t*) = *e* ^∓*jk*_*z*,*m*_^TV^ *z*^ψ_*m*_^TV^(*t*) as previously defined and indicated in Figure 1(b). In fact, the eigenfunction ψ_*m*_^TV^(*t*) is a linear combination of harmonics in {···, *e* ^*jω*_–2_ *t*^, *e* ^*jω*_–1_ *t*^, *e* ^*jω*_0_ *t*^, *e* ^*jω*_+1_ *t*^, *e* ^*jω*_+2_ *t*^, ···}, and the *m*th harmonic, i.e., *e* ^*jω*_*m*_ *t*^, is dominant (please see the Supporting Information). Thus, each eigenfunction (*m*th mode) for the TV regions would be defined as

$$\psi_{m}^{\mathrm{TV}}\left(t\right)=\underset{n}{\sum }\Psi_{m,n}^{\mathrm{TV}}e^{j\omega_{n}t}$$ 4

In the above formula, Ψ_*m*,*n*_^TV^ can be derived by dispersion analysis of the time-varying region or 3D time crystals, which depends on the modulation profile and *k*_*x*,inc_ (see the Supporting Information for the detailed formulations). The origin of the multiharmonic response here is actually the excitation of such modes. Accordingly, the multiharmonic electric field, i.e., *E*_*x*_^TV^(*x*, *z*;*t*) = ∑_*m*_ *E*_*x*,*m*_(*x*, *z*)*e* ^*jω*_*m*_ *t*^, would be obtained from Maxwell’s curl relations by extracting its harmonics (*E*_*x*,*m*_) as

$$\underset{m}{\sum }\underset{n}{\sum }j\omega_{m}\epsilon_{r,m-n}E_{x,n}e^{j\omega_{m}t}=-\frac{\partial H_{y}^{\mathrm{TV}}\left(x,z;t\right)}{\partial z}$$ 5

where ϵ_*r*,*m*–*n*_ are the Fourier coefficients of the time-modulated permittivity, i.e., ϵ_*r*_(*t*) = ∑_*m*_ϵ_*r*,*m*_ *e* ^*jm*Ω*t*^.

This framework allows us to systematically express the fields inside time-varying slabs, where interharmonic coupling arises naturally from the Fourier coefficients of the modulation profile. Differently, in static regions, the field expansions remain multiharmonic, but the harmonics are uncoupled and simply propagate independently.

For the optical device consisting of two modulated slabs, we employ this formalism to describe the fields in all regions of the structure. Specifically, expansions are written for the upper air region, the upper time-varying (TV) slab, the central hyperbolic medium composed of AZO/ZnO multilayers, the lower TV slab, and the lower air region. The hyperbolic medium itself is treated using effective medium theory (details in the Supporting Information), while the TV slabs are modeled using the harmonic expansions in eqs 3 and 5. By enforcing boundary conditions on the tangential field components across all four interfaces, a complete set of coupled equations for the modal coefficients in each region is obtained. A compact block-matrix formulation of this problem is derived and presented in the Supporting Information, providing a concrete framework for computing reflection and transmission coefficients for both forward and backward incidence.

A similar procedure is followed for the microwave device, where the time variation is confined to thin resistive metasurfaces rather than volumetric slabs. In this case, the fields in the surrounding static regions (air and wire medium) are expanded in multiharmonic form, but, as in the optical case, the harmonics are uncoupled in the static domains. The coupling arises solely at the interfaces, where the sheet conductance σ­(*t*) is sinusoidally modulated.

At a sheet with temporally varying conductance σ­(*t*), the boundary condition, which is *H*_*y*_^up^ – *H*_*y*_^down^ = *J*_*s*_(*t*) = σ­(*t*)*E*_*x*_(*t*), couples harmonics via convolution. Expanding σ­(*t*) and *E*_*x*_(*t*) into Fourier series, we obtain

$$\underset{m}{\sum }\left(H_{y,m}^{\mathrm{up}}-H_{y,m}^{\mathrm{down}}\right)e^{j\omega_{m}t}=\underset{m}{\sum }\left(\underset{n}{\sum }\sigma_{n}E_{x,m-n}\right)e^{j\omega_{m}t}$$ 6

Equation 6 can be written compactly as a Toeplitz matrix acting on the vector of harmonic fields, making explicit how each harmonic at ω_*m*_ couples to its neighbors ω_*m* ± 1_ through the Fourier coefficients σ_*n*_ of the modulation profile.

Accordingly, the full system for the microwave implementation consists of three regions separated by two time-modulated boundaries: the upper air domain, the upper time-modulated sheet, the hyperbolic wire medium, the lower time-modulated sheet, and the lower air domain. The wire medium itself is homogenized using a Drude-type effective permittivity along the wire axis, while supporting hyperbolic dispersion in the transverse plane.^41^ As before, continuity of tangential electric fields across the time-varying sheets is enforced, but for the magnetic fields, surface-current terms induce multiharmonic discontinuities. This leads to a coupled set of equations relating reflection, transmission, and internal modal coefficients across all harmonics. A compact block-matrix formulation of this system is also provided in the Supporting Information, in direct analogy to the slab-based optical case.

### Numerical Simulation

To verify our theoretical analysis, we employed full-wave simulations. Typical FEM solvers that compute the electromagnetic response in the frequency domain do not inherently support the simulation of time-varying configurations, and additional setups are required to simulate a time-varying structure accurately. Since our modulation scheme is periodic and even harmonic (sinusoidal), we expected to observe multiharmonic frequency responses in every region. Thus, for such a scenario, it is more efficient to simulate the problem in the frequency domain rather than the time domain, e.g., the finite difference time domain (FDTD) method. Therefore, we have chosen FEM solvers. It is evident that FEM solvers can simulate geometrically complex time-varying problems more effectively than other frequency-domain methods, such as the finite difference frequency domain (FDFD),^43^ making this type of solver a reasonable choice for customization and development, thanks to the conformal meshing capabilities in FEM. Additionally, unlike previous studies where numerical simulations and postprocessing relied on slow (adiabatic) temporal modulations, the multiharmonic coupled FEM solvers used here can accurately capture the system’s response under fast modulation conditions.^17,44^ Consequently, this simulation framework eliminates the need for temporal adiabatic assumptions, ensuring correct and stable results even when the modulation frequency is high.

As mentioned previously, we must implement additional setups in a typical or commercial FEM solver to carry out a full-wave simulation for time-varying (time-modulated) configurations. In this fashion, we should consider an FEM solver for each of the harmonics we are interested in to find the response. The pivotal step is to couple all of these FEM solvers together (Harmonic-Balance FEM) based on the modulation profile and the time-varying regions. To be more specific, for the first optical design, we had time-modulated slabs (3D time crystals), which are in fact regions or domains whose permittivity is modulated. In this setup, the time modulations induce volumetric polarizations at adjacent harmonics of each harmonic ω_*m*_, i.e., ω_*m*_ ± Ω, whose amplitude and phase are determined based on the modulation profile of that region. Likewise, for the second microwave configuration, including time-modulated surfaces or sheets (2D time crystals), the surface conductance of the sheets is modulated. In this case, the temporal variation of the sheet conductance generates surface currents at neighboring harmonics, shifted by ± Ω from each ω_*m*_, with their strengths determined by the modulation waveform imposed on the interface. The coupled FEM solvers in both configurations (optical and microwave designs) can compute the multiharmonic response. In such a tailored simulation setup, the computational dimension of the full-wave simulation is folded by the number of harmonics studied through the coupled solvers. Importantly, our simulation results show excellent agreement with the analytical approach described earlier. In particular, the harmonic spectra plotted in Figures 2(c) and 4(c) show perfect agreement between theory and simulation for the optical and microwave devices, respectively, validating the accuracy of both methods.

## Supplementary Material







## Notes

Theoretical analysis, additional derivations, expanded performance analysis, pump–field power-exchange calculations, and supplementary figures (PDF)
Time-domain evolution of the magnetic-field component at the operating frequency under both forward and backward illumination of the optical device (MP4)
Time-domain evolution of the magnetic-field component at the operating frequency under both forward and backward illumination of the microwave device (MP4)
