# Supplementary material for: Nonreciprocal Negative Refraction Enabled by Photonic Time Crystals
Source: Nano Lett. 2026 Jan 23;26(4):1569–77. doi: 10.1021/acs.nanolett.5c06214 (PMC12879925; doi:10.1021/acs.nanolett.5c06214)
Supplement: Supplementary file 1 [file nl5c06214_si_001.pdf]

Supporting Information

Nonreciprocal Negative Refraction Enabled by Photonic Time Crystals

Mohammad R. Tavakol[1] and Wenshan Cai[1,2,*]

[1]School of Electrical and Computer Engineering, Georgia Institute of Technology, Atlanta, Georgia 30332, United States

[2]School of Materials Science and Engineering, Georgia Institute of Technology, Atlanta, Georgia 30332, United States

*Email: wcai@gatech.edu

Contents

Optical configuration **2**

Matrix formulation for time-modulated-media dispersion 2

TE-polarized extension: Matrix formulation for time-modulated-media dispersion . . . 4

Analysis of the multilayer structure composed of two modulated slabs surrounding a hyperbolic medium 7

Performance analysis of the optical device in more detail 11

Power exchange calculations in the presence of time-varying media 13

Microwave configuration **16**

Matrix formulation for a hyperbolic medium covered by two time-modulated conductive sheets 16

Performance analysis of the microwave device in more detail 20

Power exchange calculations in the presence of time-varying conductive sheets 22

Optical configuration

Matrix formulation for time-modulated-media dispersion

We derive here the dispersion relation for transverse–magnetic (TM) waves in a dielectric whose relative permittivity varies periodically in time. Starting from Maxwell's curl equations,

$$\nabla \times \mathbf{E}(t) = -\mu_0 \frac{\partial \mathbf{H}(t)}{\partial t}, \qquad \nabla \times \mathbf{H}(t) = \frac{\partial}{\partial t}[\epsilon_0\, \epsilon_r(t)\mathbf{E}(t)]\,,$$

and eliminating $\mathbf{E}(t)$, we obtain the magnetic–field wave equation

$$\nabla^2 \mathbf{H}(t) - \frac{1}{c^2}\frac{\partial}{\partial t}\left[\epsilon_r(t)\frac{\partial \mathbf{H}(t)}{\partial t}\right] = 0. \tag{S1}$$

We focus on TM polarization and assume spatial invariance along y, so that

$$\mathbf{H}(x, z, t) = H_y(x, z, t)\,\hat{\mathbf{y}}.$$

The temporally periodic relative permittivity is expanded as

$$\epsilon_r(t) = \sum_{n\in\mathbb{Z}} \epsilon_{r,n}\, e^{jn\Omega t}, \qquad T = \frac{2\pi}{\Omega},$$

and the magnetic field admits the Floquet expansion

$$H_y(x, z, t) = e^{-jk_x x} \sum_{m\in\mathbb{Z}} H_m(z)\, e^{j(\omega_0 + m\Omega)t}.$$

Substituting these series into Eq. (S1) and matching coefficients of $e^{j(\omega_0+m\Omega)t}$ gives the coupled-harmonic relation

$$\left(k_x^2 + k_{z,m}^2\right) H_m = \frac{1}{c^2}\sum_{n\in\mathbb{Z}} \epsilon_{r,n}\,(\omega_0 + m\Omega)(\omega_0 + (m-n)\Omega)\, H_{m-n}. \tag{S2}$$

where $k_{z,m}$ is the longitudinal wavenumber of the mth harmonic.

Compact matrix representation. We define the multiharmonic amplitude vector

$$\underline{H} = (\ldots, H_{-1}, H_0, H_{+1}, \ldots)^{\mathsf{T}},$$

the diagonal operator

$$\underline{\underline{k_z}}^2 = \mathrm{diag}(\ldots, k_{z,-1}^2, k_{z,0}^2, k_{z,+1}^2, \ldots),$$

and the diagonal frequency operator

$$\underline{\underline{\omega}} = \mathrm{diag}(\ldots, \omega_{-1}, \omega_0, \omega_{+1}, \ldots), \qquad \omega_m = \omega_0 + m\Omega.$$

The Toeplitz permittivity operator is written as

$$\underline{\underline{\epsilon_r}} = \begin{pmatrix} \ddots & \vdots & \vdots & \vdots & \iddots \\ \cdots & \epsilon_{r,0} & \epsilon_{r,-1} & \epsilon_{r,-2} & \cdots \\ \cdots & \epsilon_{r,+1} & \epsilon_{r,0} & \epsilon_{r,-1} & \cdots \\ \cdots & \epsilon_{r,+2} & \epsilon_{r,+1} & \epsilon_{r,0} & \cdots \\ \iddots & \vdots & \vdots & \vdots & \ddots \end{pmatrix}.$$

Equation (S2) becomes

$$\left(k_x^2 \underline{\underline{I}} + \underline{\underline{k_z}}^2\right) \underline{H} = \frac{1}{c^2} \underline{\underline{\omega}}\, \underline{\underline{\epsilon_r}}\, \underline{\underline{\omega}}\, \underline{H},$$

which yields the generalized eigenvalue problem

$$\left[k_x^2 \underline{\underline{I}} + \underline{\underline{k_z}}^2 - \frac{1}{c^2} \underline{\underline{\omega}}\, \underline{\underline{\epsilon_r}}\, \underline{\underline{\omega}}\right] \underline{H} = 0. \tag{S3}$$

Nontrivial solutions require the dispersion condition

$$\det\left(k_x^2 \underline{\underline{I}} + \underline{\underline{k_z}}^2 - \frac{1}{c^2} \underline{\underline{\omega}}\, \underline{\underline{\epsilon_r}}\, \underline{\underline{\omega}}\right) = 0. \tag{S4}$$

Electric field reconstruction. Once the eigenpair $(k_{z,m}, \underline{H})$ is determined, the harmonic electric-field coefficients follow from Maxwell's equations:

$$E_m(x,z) = \epsilon_0^{-1}\, \underline{\underline{\epsilon_r}}^{-1} \left(\frac{k_{z,m}}{\omega_m}\hat{\mathbf{x}} - \frac{k_x}{\omega_m}\hat{\mathbf{z}} \right) H_m,$$

and the total field is reconstructed as

$$\mathbf{E}(x,z,t) = \sum_{m\in\mathbb{Z}} E_m(x,z)\, e^{j(\omega_0 + m\Omega)t}.$$

Figure S1 illustrates representative multiharmonic eigenmodes obtained from the dispersion equation (S3). Each eigenmode of the time-varying slab is represented by the column vector $\underline{\Psi}_m^{\mathrm{TV}}$, whose elements give the complex amplitudes of the harmonics $\{\ldots,\, e^{j\omega_{-1}t},\, e^{j\omega_0 t},\, e^{j\omega_{+1}t}, \ldots\}$ that collectively form the mth Floquet eigenmode. The full eigenfunction matrix

$$\underline{\underline{\Psi}}^{\mathrm{TV}} = \left[\ldots, \underline{\Psi}_{-2}^{\mathrm{TV}}, \underline{\Psi}_{-1}^{\mathrm{TV}}, \underline{\Psi}_{0}^{\mathrm{TV}}, \underline{\Psi}_{+1}^{\mathrm{TV}}, \underline{\Psi}_{+2}^{\mathrm{TV}}, \ldots\right]$$

is therefore constructed by stacking these modal vectors as its columns. As seen in Fig. S1(b), the relative level of the side harmonics grows progressively as we move from the mode with index $m = -2$ toward $m = +2$, indicating that the temporal modulation induces slightly stronger harmonic mixing for the positive-index modes. This trend reflects the asymmetric coupling among Floquet orders introduced by the time modulation and quantitatively captured by the matrix formulation above.

TE-polarized extension: Matrix formulation for time-modulated-media dispersion

We derive here the dispersion relation for transverse–electric (TE) waves in a dielectric whose relative permittivity varies periodically in time. Starting from Maxwell's curl equations,

$$\nabla \times \mathbf{E}(t) = -\mu_0 \frac{\partial \mathbf{H}(t)}{\partial t}, \qquad \nabla \times \mathbf{H}(t) = \frac{\partial}{\partial t}\left[\epsilon_0\, \epsilon_r(t)\mathbf{E}(t)\right],$$

and eliminating $\mathbf{H}(t)$, we obtain the electric–field wave equation

$$\nabla^2\mathbf{E}(t) - \frac{1}{c^2}\,\epsilon_r(t)\,\frac{\partial^2\mathbf{E}(t)}{\partial t^2} = 0. \tag{S5}$$

We focus on TE polarization and assume spatial invariance along y, so that

$$\mathbf{E}(x,z,t) = E_y(x,z,t)\,\hat{\mathbf{y}}.$$

The temporally periodic relative permittivity is expanded as

$$\epsilon_r(t) = \sum_{n\in\mathbb{Z}} \epsilon_{r,n}\, e^{jn\Omega t}, \qquad T = \frac{2\pi}{\Omega},$$

and the electric field admits the Floquet expansion

$$E_y(x,z,t) = e^{-jk_x x}\sum_{m\in\mathbb{Z}} E_m(z)\, e^{j(\omega_0+m\Omega)t}.$$

Substituting these series into Eq. (S5) and matching coefficients of $e^{j(\omega_0+m\Omega)t}$ gives the coupled-harmonic relation

$$\left(k_x^2 + k_{z,m}^2\right)E_m = \frac{1}{c^2}\sum_{n\in\mathbb{Z}} \epsilon_{r,n}\left(\omega_0 + (m-n)\Omega\right)^2 E_{m-n}, \tag{S6}$$

where $k_{z,m}$ is the longitudinal wavenumber of the mth harmonic.

Compact matrix representation. We define the multiharmonic amplitude vector

$$\underline{E} = (\ldots, E_{-1}, E_0, E_{+1}, \ldots)^{\mathsf{T}},$$

the diagonal operator

$$\underline{\underline{k_z}}^2 = \mathrm{diag}(\ldots, k_{z,-1}^2, k_{z,0}^2, k_{z,+1}^2, \ldots),$$

and the diagonal frequency operator

$$\underline{\underline{\omega}} = \mathrm{diag}(\ldots, \omega_{-1}, \omega_0, \omega_{+1}, \ldots), \qquad \omega_m = \omega_0 + m\Omega.$$

The Toeplitz permittivity operator is written as

$$\underline{\underline{\epsilon_r}} = \begin{pmatrix} \ddots & \vdots & \vdots & \vdots & \iddots \\ \cdots & \epsilon_{r,0} & \epsilon_{r,-1} & \epsilon_{r,-2} & \cdots \\ \cdots & \epsilon_{r,+1} & \epsilon_{r,0} & \epsilon_{r,-1} & \cdots \\ \cdots & \epsilon_{r,+2} & \epsilon_{r,+1} & \epsilon_{r,0} & \cdots \\ \iddots & \vdots & \vdots & \vdots & \ddots \end{pmatrix}.$$

Equation (S6) becomes

$$\left(k_x^2\,\underline{\underline{I}} + \underline{\underline{k_z}}^2\right)\underline{E} = \frac{1}{c^2}\,\underline{\underline{\epsilon_r}}\,\underline{\underline{\omega}}^2\,\underline{E},$$

which yields the generalized eigenvalue problem

$$\left[k_x^2\,\underline{\underline{I}} + \underline{\underline{k_z}}^2 - \frac{1}{c^2}\,\underline{\underline{\epsilon_r}}\,\underline{\underline{\omega}}^2\right]\underline{E} = 0. \tag{S7}$$

Nontrivial solutions require the dispersion condition

$$\det\left(k_x^2\,\underline{\underline{I}} + \underline{\underline{k_z}}^2 - \frac{1}{c^2}\,\underline{\underline{\epsilon_r}}\,\underline{\underline{\omega}}^2\right) = 0. \tag{S8}$$

Magnetic field reconstruction. Once the eigenpair $(k_{z,m}, \underline{E})$ is determined, the harmonic magnetic-field coefficients follow from Maxwell's equations:

$$\mathbf{H}_m(x,z) = \left(\frac{k_{z,m}}{\mu_0\,\omega_m}\hat{\mathbf{x}} - \frac{k_x}{\mu_0\,\omega_m}\hat{\mathbf{z}}\right) E_m(z)\,e^{-jk_x x},$$

and the total field is reconstructed as

$$\mathbf{H}(x,z,t) = \sum_{m\in\mathbb{Z}} \mathbf{H}_m(x,z)\,e^{j(\omega_0 + m\Omega)t}.$$

Remark on the TM–TE correspondence. The TE formulation follows the same Floquet–Toeplitz framework as the TM case, but with a different temporal operator in the governing wave equation. Specifically, TM polarization involves the operator $\partial_t[\epsilon_r(t)\partial_t(\cdot))$, whereas TE

polarization involves $\epsilon_r(t)\partial_t^2(\cdot)$. As a result, the generalized eigenvalue problem for TE polarization is obtained from the TM counterpart by replacing the operator $\underline{\underline{\omega}}\,\underline{\underline{\epsilon_r}}\,\underline{\underline{\omega}}$ with $\underline{\underline{\epsilon_r}}\,\underline{\underline{\omega}}^2$.

Analysis of the multilayer structure composed of two modulated slabs surrounding a hyperbolic medium

We now specialize the multiharmonic formalism to the multilayer system used in the main text, consisting of two time-modulated dielectric slabs surrounding a static hyperbolic medium. The magnetic field in each region is expanded as

$$\begin{aligned}
\mathbf{H}^{\mathrm{I}}(x,z;t) &= \left[e^{-jk_{x,\mathrm{inc}}x}\,e^{-jk_{z,\mathrm{inc}}z}\,e^{j\omega_0 t} + e^{-jk_{x,\mathrm{inc}}x}\sum_m r_m\,e^{+jk_{z,m}z}e^{j\omega_m t}\right]\hat{\mathbf{y}},\\
\mathbf{H}^{\mathrm{TV1}}(x,z;t) &= e^{-jk_{x,\mathrm{inc}}x}\sum_m\left(a_m^{\mathrm{TV1}}e^{-jk_{z,m}^{\mathrm{TV1}}z} + b_m^{\mathrm{TV1}}e^{+jk_{z,m}^{\mathrm{TV1}}z}\right)\psi_m^{\mathrm{TV1}}(t)\,\hat{\mathbf{y}},\\
\mathbf{H}^{\mathrm{II}}(x,z;t) &= e^{-jk_{x,\mathrm{inc}}x}\sum_m\left(a_m e^{-jk_{z,m}^{\mathrm{hyp}}z} + b_m e^{+jk_{z,m}^{\mathrm{hyp}}z}\right)e^{j\omega_m t}\,\hat{\mathbf{y}},\\
\mathbf{H}^{\mathrm{TV2}}(x,z;t) &= e^{-jk_{x,\mathrm{inc}}x}\sum_m\left(a_m^{\mathrm{TV2}}e^{-jk_{z,m}^{\mathrm{TV2}}z} + b_m^{\mathrm{TV2}}e^{+jk_{z,m}^{\mathrm{TV2}}z}\right)\psi_m^{\mathrm{TV2}}(t)\,\hat{\mathbf{y}},\\
\mathbf{H}^{\mathrm{III}}(x,z;t) &= e^{-jk_{x,\mathrm{inc}}x}\sum_m t_m e^{-jk_{z,m}z}e^{j\omega_m t}\,\hat{\mathbf{y}},
\end{aligned}$$

where $\omega_m = \omega_0 + m\Omega$, and $\psi_m^{\mathrm{TV1}}(t)$ and $\psi_m^{\mathrm{TV2}}(t)$ are the eigenfunctions of the time-varying slabs in regions TV1 and TV2, respectively, associated with the eigenvalues $k_{z,m}^{\mathrm{TV1}}$ and $k_{z,m}^{\mathrm{TV2}}$ (see Sec. 1.1).

Layer geometry. The z–stratified architecture is

$$\begin{aligned}
z < -d - \tfrac{L}{2} &: \text{air (incidence region I)},\\
-d - \tfrac{L}{2} < z < -\tfrac{L}{2} &: \text{time-varying slab 1 (TV1)},\\
-\tfrac{L}{2} < z < \tfrac{L}{2} &: \text{static hyperbolic slab (region II)},\\
\tfrac{L}{2} < z < \tfrac{L}{2} + d &: \text{time-varying slab 2 (TV2)},\\
\tfrac{L}{2} + d < z &: \text{air (transmission region III)},
\end{aligned}$$

where d is the thickness of each modulated slab and L is the thickness of the hyperbolic core.

Multiharmonic vectors and operators. We collect the harmonic amplitudes into column vectors,

$$\begin{aligned}
&\underline{r} \triangleq (\ldots, r_{-2}, r_{-1}, r_0, r_{+1}, r_{+2}, \ldots)^{\mathsf{T}}, \quad \underline{a^{\mathrm{TV1}}} \triangleq (\ldots, a_{-2}^{\mathrm{TV1}}, a_{-1}^{\mathrm{TV1}}, a_0^{\mathrm{TV1}}, a_{+1}^{\mathrm{TV1}}, \ldots)^{\mathsf{T}}, \\
&\underline{b^{\mathrm{TV1}}},\ \underline{a},\ \underline{b},\ \underline{a^{\mathrm{TV2}}},\ \underline{b^{\mathrm{TV2}}},\ \underline{t} \quad \text{defined analogously,}
\end{aligned}$$

and define the diagonal operators

$$\begin{aligned}
&\underline{\underline{k_z}} \triangleq \mathrm{diag}(\ldots, k_{z,-2}, k_{z,-1}, k_{z,0}, k_{z,+1}, k_{z,+2}, \ldots), \quad k_{z,m} = \sqrt{\left(\tfrac{\omega_m}{c}\right)^2 - k_{x,\mathrm{inc}}^2}, \\
&\underline{\underline{k_z^{\mathrm{hyp}}}} \triangleq \mathrm{diag}(\ldots, k_{z,-2}^{\mathrm{hyp}}, k_{z,-1}^{\mathrm{hyp}}, \ldots), \quad k_{z,m}^{\mathrm{hyp}} = \sqrt{\epsilon_t\left[\left(\tfrac{\omega_m}{c}\right)^2 - \tfrac{k_{x,\mathrm{inc}}^2}{\epsilon_z}\right]}, \\
&\underline{\underline{k_z^{\mathrm{TV1}}}},\ \ \underline{\underline{k_z^{\mathrm{TV2}}}} \quad \text{(diagonal matrices of TV1/TV2 longitudinal wavenumbers),} \\
&\underline{\underline{\omega}} \triangleq \mathrm{diag}(\ldots, \omega_{-2}, \omega_{-1}, \omega_0, \omega_{+1}, \omega_{+2}, \ldots), \quad \omega_m = \omega_0 + m\Omega.
\end{aligned}$$

The temporally modulated permittivity profiles in TV1 and TV2 are represented by Toeplitz operators

$$\underline{\underline{\epsilon_r^{\mathrm{TV1}}}} \quad \text{and} \quad \underline{\underline{\epsilon_r^{\mathrm{TV2}}}},$$

whose (p, q) elements are given by the corresponding permittivity harmonic $\epsilon_{r,p-q}^{\mathrm{TV1}}$ or $\epsilon_{r,p-q}^{\mathrm{TV2}}$, respectively. We also define the unit vector

$$\underline{1_0} \triangleq (\ldots, 0, 0, 1, 0, 0, \ldots)^{\mathsf{T}},$$

with a single nonzero entry at the central (fundamental) harmonic.

Modal matrices of the time-varying slabs. For each time-varying region, the mth multiharmonic eigenmode is represented by the column vector $\underline{\Psi}_m^{\mathrm{TV1}}$ or $\underline{\Psi}_m^{\mathrm{TV2}}$, whose entries are the complex amplitudes of the harmonics $\{\ldots, e^{j\omega_{-2}t}, e^{j\omega_{-1}t}, e^{j\omega_0 t}, e^{j\omega_{+1}t}, \ldots\}$ in the eigenfunction $\psi_m^{\mathrm{TV1}}(t)$

or $\psi_m^{\mathrm{TV2}}(t)$. We collect these into the modal matrices

$$\underline{\underline{\Psi}}^{\mathrm{TV1}} \triangleq (\ldots, \underline{\Psi}_{-2}^{\mathrm{TV1}}, \underline{\Psi}_{-1}^{\mathrm{TV1}}, \underline{\Psi}_{0}^{\mathrm{TV1}}, \underline{\Psi}_{+1}^{\mathrm{TV1}}, \underline{\Psi}_{+2}^{\mathrm{TV1}}, \ldots), \quad \underline{\underline{\Psi}}^{\mathrm{TV2}} \triangleq (\ldots, \underline{\Psi}_{-2}^{\mathrm{TV2}}, \ldots, \underline{\Psi}_{+2}^{\mathrm{TV2}}, \ldots),$$

so that columns index the eigenmodes and rows index the temporal harmonics, consistent with the notation introduced in Fig. S1.

Multiharmonic magnetic fields in each region. Using the above definitions, the harmonic-domain magnetic fields in the five regions can be written compactly as

$$\begin{aligned}
\underline{\mathbf{H}}^{\mathrm{I}}(x,z) &= e^{-jk_{x,\mathrm{inc}}x}\left[e^{-jk_{z,\mathrm{inc}}z}\,\underline{1_0} + e^{+j\underline{\underline{k_z}}z}\,\underline{r}\right]\hat{\mathbf{y}},\\
\underline{\mathbf{H}}^{\mathrm{TV1}}(x,z) &= e^{-jk_{x,\mathrm{inc}}x}\,\underline{\underline{\Psi}}^{\mathrm{TV1}}\left[e^{-j\underline{\underline{k_z^{\mathrm{TV1}}}}z}\,\underline{a^{\mathrm{TV1}}} + e^{+j\underline{\underline{k_z^{\mathrm{TV1}}}}z}\,\underline{b^{\mathrm{TV1}}}\right]\hat{\mathbf{y}},\\
\underline{\mathbf{H}}^{\mathrm{II}}(x,z) &= e^{-jk_{x,\mathrm{inc}}x}\left[e^{-j\underline{\underline{k_z^{\mathrm{hyp}}}}z}\,\underline{a} + e^{+j\underline{\underline{k_z^{\mathrm{hyp}}}}z}\,\underline{b}\right]\hat{\mathbf{y}},\\
\underline{\mathbf{H}}^{\mathrm{TV2}}(x,z) &= e^{-jk_{x,\mathrm{inc}}x}\,\underline{\underline{\Psi}}^{\mathrm{TV2}}\left[e^{-j\underline{\underline{k_z^{\mathrm{TV2}}}}z}\,\underline{a^{\mathrm{TV2}}} + e^{+j\underline{\underline{k_z^{\mathrm{TV2}}}}z}\,\underline{b^{\mathrm{TV2}}}\right]\hat{\mathbf{y}},\\
\underline{\mathbf{H}}^{\mathrm{III}}(x,z) &= e^{-jk_{x,\mathrm{inc}}x}\,e^{-j\underline{\underline{k_z}}z}\,\underline{t}\,\hat{\mathbf{y}}.
\end{aligned} \tag{S9}$$

Electric fields from Maxwell's equations. We similarly collect the harmonic amplitudes of the electric-field components into $\underline{\mathbf{E}}_x$ and $\underline{\mathbf{E}}_z$. In the time-varying slabs, the multiharmonic constitutive relation yields

$$\begin{aligned}
\underline{\mathbf{E}}_x^{\mathrm{TV1,TV2}}(x,z) &= -\frac{1}{j\epsilon_0}\left(\underline{\underline{\omega}}\,\underline{\underline{\epsilon_r^{\mathrm{TV1,TV2}}}}\right)^{-1}\frac{\partial \underline{\mathbf{H}}^{\mathrm{TV1,TV2}}(x,z)}{\partial z},\\
\underline{\mathbf{E}}_z^{\mathrm{TV1,TV2}}(x,z) &= \frac{1}{j\epsilon_0}\left(\underline{\underline{\omega}}\,\underline{\underline{\epsilon_r^{\mathrm{TV1,TV2}}}}\right)^{-1}\frac{\partial \underline{\mathbf{H}}^{\mathrm{TV1,TV2}}(x,z)}{\partial x},
\end{aligned} \tag{S10}$$

where the differential operators act element-wise on the z-dependent factors in Eq. (S9).

For the static regions, it is convenient to introduce the diagonal matrix of total wavenumbers

$$\underline{\underline{k}} \triangleq \mathrm{diag}(\ldots, k_{-2}, k_{-1}, k_0, k_{+1}, k_{+2}, \ldots), \qquad k_m = \frac{\omega_m}{c},$$

and the diagonal propagation-angle matrix

$$\underline{\underline{\theta}} \triangleq \mathrm{diag}(\ldots, \theta_{-2}, \theta_{-1}, \theta_0, \theta_{+1}, \theta_{+2}, \ldots), \qquad \theta_m = \cos^{-1}\!\left(\frac{k_{z,m}}{k_m}\right),$$

together with their element-wise cosine and sine matrices,

$$\cos\underline{\underline{\theta}} \triangleq \mathrm{diag}(\ldots, \cos\theta_{-2}, \ldots, \cos\theta_{+2}, \ldots), \quad \sin\underline{\underline{\theta}} \triangleq \mathrm{diag}(\ldots, \sin\theta_{-2}, \ldots, \sin\theta_{+2}, \ldots).$$

Using the usual TM relations, the x–component of the electric field in each region can then be expressed as

$$\begin{aligned}
\underline{\mathbf{E}}_x^{\mathrm{I}}(x,z) &= \eta\, e^{-jk_{x,\mathrm{inc}}x}\left[\cos\theta_{\mathrm{inc}}\, e^{-jk_{z,\mathrm{inc}}z}\, \underline{1_0} - \cos\underline{\underline{\theta}}\, e^{+j\underline{\underline{k_z}}z}\, \underline{r}\right],\\
\underline{\mathbf{E}}_x^{\mathrm{TV1}}(x,z) &= \epsilon_0^{-1}\, e^{-jk_{x,\mathrm{inc}}x}\left(\underline{\underline{\omega}}\, \underline{\underline{\epsilon_r^{\mathrm{TV1}}}}\right)^{-1} \underline{\underline{\Psi}}^{\mathrm{TV1}}\, \underline{\underline{k_z^{\mathrm{TV1}}}}\left[e^{-j\underline{\underline{k_z^{\mathrm{TV1}}}}z}\, \underline{a^{\mathrm{TV1}}} - e^{+j\underline{\underline{k_z^{\mathrm{TV1}}}}z}\, \underline{b^{\mathrm{TV1}}}\right],\\
\underline{\mathbf{E}}_x^{\mathrm{II}}(x,z) &= \eta\, e^{-jk_{x,\mathrm{inc}}x}\, \underline{\underline{k_z^{\mathrm{hyp}}}}\, \underline{\underline{k}}^{-1}\left[e^{-j\underline{\underline{k_z^{\mathrm{hyp}}}}z}\, \underline{a} - e^{+j\underline{\underline{k_z^{\mathrm{hyp}}}}z}\, \underline{b}\right]\frac{1}{\epsilon_t},\\
\underline{\mathbf{E}}_x^{\mathrm{TV2}}(x,z) &= \epsilon_0^{-1}\, e^{-jk_{x,\mathrm{inc}}x}\left(\underline{\underline{\omega}}\, \underline{\underline{\epsilon_r^{\mathrm{TV2}}}}\right)^{-1} \underline{\underline{\Psi}}^{\mathrm{TV2}}\, \underline{\underline{k_z^{\mathrm{TV2}}}}\left[e^{-j\underline{\underline{k_z^{\mathrm{TV2}}}}z}\, \underline{a^{\mathrm{TV2}}} - e^{+j\underline{\underline{k_z^{\mathrm{TV2}}}}z}\, \underline{b^{\mathrm{TV2}}}\right],\\
\underline{\mathbf{E}}_x^{\mathrm{III}}(x,z) &= \eta\, e^{-jk_{x,\mathrm{inc}}x}\, \cos\underline{\underline{\theta}}\, e^{-j\underline{\underline{k_z}}z}\, \underline{t}.
\end{aligned}$$

Boundary conditions at the interfaces

The four interfaces are located at

$$z_1 = -d - \frac{L}{2}, \qquad z_2 = -\frac{L}{2}, \qquad z_3 = \frac{L}{2}, \qquad z_4 = \frac{L}{2} + d.$$

At each interface, we enforce continuity of the tangential fields H_y and E_x. Evaluating Eqs. (S9) and the above expressions for $\underline{\mathbf{E}}_x$ at $z = z_i$ leads to a set of linear relations among the unknown coefficient vectors $\underline{r}$, $\underline{a^{\mathrm{TV1}}}$, $\underline{b^{\mathrm{TV1}}}$, $\underline{a}$, $\underline{b}$, $\underline{a^{\mathrm{TV2}}}$, $\underline{b^{\mathrm{TV2}}}$, and $\underline{t}$. For example, at the first interface $z = z_1$

(between regions I and TV1) we obtain

$$
\begin{aligned}
e^{-jk_{z,\mathrm{inc}}z_1}\,\underline{1_0} + e^{+j\underline{\underline{k_z}}z_1}\,\underline{r} &= \underline{\underline{\Psi}}^{\mathrm{TV1}}\Big[e^{-j\underline{\underline{k_z^{\mathrm{TV1}}}}z_1}\,\underline{a^{\mathrm{TV1}}} + e^{+j\underline{\underline{k_z^{\mathrm{TV1}}}}z_1}\,\underline{b^{\mathrm{TV1}}}\Big],\\
\eta\Big[\cos\theta_{\mathrm{inc}}\,e^{-jk_{z,\mathrm{inc}}z_1}\,\underline{1_0} - \cos\underline{\underline{\theta}}\,e^{+j\underline{\underline{k_z}}z_1}\,\underline{r}\Big] &= \epsilon_0^{-1}\Big(\underline{\underline{\omega}}\,\underline{\underline{\epsilon_r^{\mathrm{TV1}}}}\Big)^{-1}\,\underline{\underline{\Psi}}^{\mathrm{TV1}}\,\underline{\underline{k_z^{\mathrm{TV1}}}}\Big[e^{-j\underline{\underline{k_z^{\mathrm{TV1}}}}z_1}\,\underline{a^{\mathrm{TV1}}} - e^{+j\underline{\underline{k_z^{\mathrm{TV1}}}}z_1}\,\underline{b^{\mathrm{TV1}}}\Big].
\end{aligned}
$$

Analogous pairs of continuity equations are obtained at $z = z_2$ (TV1–hyperbolic), $z = z_3$ (hyperbolic–TV2), and $z = z_4$ (TV2–air), by substituting the corresponding field expressions in regions II, TV2, and III. Collectively, these boundary conditions form a multiharmonic linear system for the unknown vectors $\underline{r}$, $\underline{a^{\mathrm{TV1}}}$, $\underline{b^{\mathrm{TV1}}}$, $\underline{a}$, $\underline{b}$, $\underline{a^{\mathrm{TV2}}}$, $\underline{b^{\mathrm{TV2}}}$, and $\underline{t}$, which is solved numerically in our simulations.

Performance analysis of the optical device in more detail

Figure S2 provides additional insight into the multiharmonic polarization generated inside the time-varying dielectric slabs. Because the relative permittivity is periodically modulated,

$$
\epsilon_r(t) = \sum_n \epsilon_{r,n} e^{jn\Omega t} \quad \overset{\mathcal{F}}{\longleftrightarrow} \quad \epsilon_r(\omega) = 2\pi \sum_n \epsilon_{r,n}\delta(\omega - n\Omega),
$$

the material response consists of discrete spectral lines spaced by the modulation frequency. The induced polarization follows

$$
\mathbf{P}(t) = \epsilon_0\,\epsilon_r(t)\mathbf{E}(t) \quad \overset{\mathcal{F}}{\longleftrightarrow} \quad \mathbf{P}(\omega) = 2\pi\epsilon_0 \sum_n \epsilon_{r,n}\mathbf{E}(\omega - n\Omega),
$$

which reflects the multiply–convolve property of Fourier transforms. Writing the fields in the Floquet form,

$$
\mathbf{E}(t) = \sum_m \mathbf{E}_m e^{jm\Omega t}, \qquad \mathbf{P}(t) = \sum_m \mathbf{P}_m e^{jm\Omega t},
$$

gives

$$
\mathbf{P}_m = \epsilon_0 \sum_n \epsilon_{r,n}\mathbf{E}_{m-n}.
$$

This relation explains the trend shown in Fig. S2: the modulation redistributes energy among neighboring sidebands, and the relative gap between the principal and side harmonics slowly decreases as we move from lower-order to higher-order eigenmodes, consistent with the modal structure illustrated in Fig. S1(b) previously.

Figures S3 and S4 further quantify how electromagnetic power is distributed among Floquet harmonics within the multilayer device. The incident power per simulation period is normalized to $P_{\text{inc}} = 1$ W/m, and all computed values are referenced to this level. These results show that temporal modulation continuously feeds energy from the fundamental harmonic into multiple sidebands, while the hyperbolic slab, being dispersive, selectively enhances or suppresses specific harmonics depending on their longitudinal wavenumbers. As a result, the internal power flow strongly depends on the incidence direction, which is essential for the nonreciprocal transmission observed in the main text.

Supplementary Video 1 visualizes the time-domain evolution of the magnetic-field component $H_y(x, z, t)$ at the operating frequency ω_0, corresponding to the zeroth-order Floquet harmonic, under both forward and backward illumination of the optical device. To enhance the perceptibility of directional differences in transmitted amplitude, the video employs a nonlinear μ-law colormap with $\mu = 10$. This dynamic-range compression makes the contrast between forward and backward transmission more visible than a linear scale would allow. The video therefore complements Figs. S3 and S4 by providing a continuous-time depiction of how the main harmonic propagates through the modulated slabs and hyperbolic core, revealing the asymmetric field amplitudes associated with nonreciprocal transmission.

Figure S5 highlights the spatial field profiles associated with forward and backward illumination, again assuming an incident power of $P_{\text{inc}} = 1$ W/m. The key observation is that the transmitted beam bends toward the "negative" side of the normal, confirming the occurrence of negative refraction in the presence of the temporal modulation. In addition, the forward and backward incidence panels show distinctly different refracted trajectories and field intensities. This directional asymmetry arises from the time modulation, which breaks time-reversal symmetry and leads to different modal interference pathways when the excitation direction is reversed.

Finally, Fig. S6 investigates the dependence of device performance on the modulation phase difference $\phi_2 - \phi_1$ applied to the two time-varying slabs. The insertion loss is defined as

$$\mathrm{IL} = -10\log_{10}(T_f),$$

with T_f denoting the forward transmittance. As shown in Fig. S6(a), the isolation is maximized at

$$\phi_2 - \phi_1 \approx \frac{\pi}{2} + \frac{\pi}{50}.$$

although the insertion loss is not minimized. Thus, we can more clearly feel the trade-off between isolation and insertion loss. This optimal point lies slightly above the $\pi/2$ quadrature condition predicted by ideal temporal coupled-mode theory.[S1] The shift arises from finite-thickness effects and the multimode interaction mediated through the dispersive hyperbolic core, which introduce additional internal phase accumulation. Consequently, the effective interference condition governing nonreciprocal transmission is achieved at a phase that is marginally offset from the idealized resonance limit.

Power exchange calculations in the presence of time-varying media

We begin with the definition of the instantaneous Poynting vector, $\mathbf{S} = \mathbf{E}\times\mathbf{H}$, and apply the vector identity $\nabla\cdot(\mathbf{E}\times\mathbf{H}) = \mathbf{H}\cdot(\nabla\times\mathbf{E}) - \mathbf{E}\cdot(\nabla\times\mathbf{H})$. Using Maxwell's curl equations, $\nabla\times\mathbf{E} = -\partial_t\mathbf{B}$ and $\nabla\times\mathbf{H} = \mathbf{J}_{\mathrm{free}} + \partial_t\mathbf{D}$, we obtain

$$\nabla\cdot\mathbf{S} + \mathbf{E}\cdot\partial_t\mathbf{D} + \mathbf{H}\cdot\partial_t\mathbf{B} = -\,\mathbf{E}\cdot\mathbf{J}_{\mathrm{free}}. \tag{S11}$$

Assuming an instantaneous scalar material law $\mathbf{D} = \epsilon(t)\mathbf{E}$, we may evaluate

$$\mathbf{E}\cdot\partial_t\mathbf{D} = \mathbf{E}\cdot\Big[\dot{\epsilon}(t)\mathbf{E} + \epsilon(t)\partial_t\mathbf{E}\Big].$$

Meanwhile,

$$\frac{\partial}{\partial t}\Big(\tfrac{1}{2}\,\mathbf{E}\cdot\mathbf{D}\Big) = \tfrac{1}{2}\,\mathbf{E}\cdot\dot{\epsilon}(t)\mathbf{E} + \mathbf{E}\cdot\epsilon(t)\partial_t\mathbf{E}.$$

Subtracting the latter from the former yields the important identity

$$\mathbf{E}\cdot \partial_t \mathbf{D} = \frac{\partial}{\partial t}\Big(\tfrac{1}{2}\,\mathbf{E}\cdot \mathbf{D}\Big) + \tfrac{1}{2}\,\mathbf{E}\cdot \dot{\epsilon}(t)\mathbf{E},$$

which shows that $\mathbf{E}\cdot \partial_t \mathbf{D}$ decomposes into a stored-energy term and an extra term that appears only when the permittivity is explicitly time dependent. Inserting this result into (S11) gives the generalized Poynting theorem for a time-varying dielectric:

$$\nabla\cdot \mathbf{S} + \frac{\partial}{\partial t}\Big(\tfrac{1}{2}\,\mathbf{E}\cdot \mathbf{D} + \tfrac{1}{2}\,\mathbf{H}\cdot \mathbf{B}\Big) = -\,\mathbf{E}\cdot \mathbf{J}_{\text{free}} - \tfrac{1}{2}\,\dot{\epsilon}(t)\,|\mathbf{E}(t)|^2. \tag{S12}$$

The final term on the right represents the per-unit-volume pump-field power exchange

$$p_\epsilon(t) \;\triangleq\; \tfrac{1}{2}\,\dot{\epsilon}(t)\,|\mathbf{E}(t)|^2,$$

which acts as a source (if positive) or sink (if negative) depending on whether energy is being injected into or extracted from the field by the modulation.

Fourier-series evaluation of the time-averaged power exchange

To compute the time-averaged pump–field power transfer over one modulation period $T = 2\pi/\Omega$, we expand the field and material modulation in their Floquet–Fourier series. The electric field is written as the real part of its multiharmonic expansion,

$$\mathbf{E}(t) = \Re\Bigg\{\sum_{m\in\mathbb{Z}} \mathbf{E}_m\, e^{j(\omega_0+m\Omega)t}\Bigg\} = \tfrac{1}{2}\sum_m \Big(\mathbf{E}_m e^{j(\omega_0+m\Omega)t} + \mathbf{E}_m^{*} e^{-j(\omega_0+m\Omega)t}\Big).$$

Averaging $|\mathbf{E}(t)|^2$ over the fast optical oscillations removes all $2\omega_0$ terms and produces a slowly varying envelope,

$$|\mathbf{E}(t)|^2 \approx \tfrac{1}{2}\sum_k \Re\Big(C_k e^{jk\Omega t}\Big), \qquad C_k = \sum_n \mathbf{E}_{n+k}\cdot \mathbf{E}_n^{*}.$$

For a single-tone permittivity modulation,

$$\epsilon(t) = \epsilon_{r,0} + \Delta\epsilon \cos(\Omega t + \phi),$$

we obtain

$$\dot{\epsilon}(t) = \Re\Big\{A_{+1}e^{j\Omega t} + A_{-1}e^{-j\Omega t}\Big\}, \qquad A_{+1} = \frac{j\Omega\Delta\epsilon}{2}e^{j\phi}, \quad A_{-1} = A_{+1}^{*}.$$

Multiplying the series for $\dot{\epsilon}(t)$ and $|\mathbf{E}(t)|^2$ and retaining only the zero-frequency (DC) term gives the period-averaged pump power transfer,

$$\langle p_\epsilon\rangle = \tfrac{1}{4}\,\Re\Big(A_{+1}C_{-1} + A_{-1}C_{+1}\Big) = \frac{\Omega\Delta\epsilon}{4}\Im\Big\{e^{-j\phi}C_{+1}\Big\}.$$

Since $C_{+1} = \sum_n \mathbf{E}_{n+1}\cdot\mathbf{E}_n^*$, we arrive at the compact form

$$\langle p_\epsilon\rangle = \frac{\Omega\Delta\epsilon}{4}\sum_{n\in\mathbb{Z}}\Im\Big\{e^{-j\phi}\,\mathbf{E}_{n+1}\cdot\mathbf{E}_n^*\Big\}.$$

Thus, for a single-tone temporal modulation, only adjacent harmonic pairs $(n, n+1)$ contribute to the cycle-averaged pump–field energy exchange, and the net transfer depends on the relative phase $e^{-j\phi}$ and the quadrature relation between $\mathbf{E}_{n+1}$ and $\mathbf{E}_n$. A positive value of $\langle p_\epsilon\rangle$ corresponds to power delivered from the modulation to the field, while a negative value indicates net extraction of power from the field by the modulation.

Figure S7 provides a direct illustration of these multiharmonic power-exchange mechanisms inside the time-varying slabs. In Fig. S7(a), the two upper panels show the individual contributions from the first and second modulated slabs (TV1 and TV2), plotted as the integrated pump–field interaction power within each slab. The bottom panel of Fig. S7(a) displays the resulting *net* interaction power, obtained by summing the contributions of the two slabs. The results reveal that the overall pump–field interaction is different for forward and backward illumination, reflecting the direction-dependent harmonic content and modal interference entering each slab, even though the difference is not strongly asymmetric.

Figure S7(b) (right panels) complements this picture by presenting the spatial distribution of

the local power-exchange density $p_\epsilon(x,z)$ across the structure. These maps show where, within the slabs, energy is transferred to or from the electromagnetic field for representative detunings $\Delta\omega/\omega_0 = \{-10\%,\, 0,\, +10\%\}$. The spatial patterns vary with both detuning and illumination direction, consistent with the multiharmonic coupling terms $\mathbf{E}_{n+1}\cdot\mathbf{E}_n^*$ that determine $\langle p_\epsilon\rangle$. Together, the panels of Fig. S7 visualize how temporal modulation redistributes power among harmonics and across space, and how this redistribution differs for opposite propagation directions in the device.

Microwave configuration

Matrix formulation for a hyperbolic medium covered by two time-modulated conductive sheets

We consider a hyperbolic slab of thickness L occupying $0 < z < L$, with relative permittivity tensor

$$\underline{\underline{\epsilon_r}} = \begin{pmatrix} \epsilon_t & 0 & 0 \\ 0 & \epsilon_t & 0 \\ 0 & 0 & \epsilon_z \end{pmatrix}.$$

Regions I ($z < 0$) and III ($z > L$) are free space. A TM-polarized plane wave is incident from region I with angular frequency ω_0 and incidence angle θ_{inc}.

The total y-polarized magnetic field in each region is expanded in Floquet harmonics at $\omega_m = \omega_0 + m\Omega$ as

$$\mathbf{H}^{\text{I}}(x,z;t) = \left[e^{-jk_{x,\text{inc}}x}e^{-jk_{z,\text{inc}}z}e^{j\omega_0 t} + e^{-jk_{x,\text{inc}}x}\sum_m r_m e^{+jk_{z,m}z}e^{j\omega_m t}\right]\hat{\mathbf{y}},$$

$$\mathbf{H}^{\text{II}}(x,z;t) = e^{-jk_{x,\text{inc}}x}\sum_m\left(a_m e^{-jk_{z,m}^{\text{hyp}}z} + b_m e^{+jk_{z,m}^{\text{hyp}}z}\right)e^{j\omega_m t}\,\hat{\mathbf{y}},$$

$$\mathbf{H}^{\text{III}}(x,z;t) = e^{-jk_{x,\text{inc}}x}\sum_m t_m e^{-jk_{z,m}z}e^{j\omega_m t}\,\hat{\mathbf{y}},$$

where

$$k_m = \frac{\omega_m}{c}, \qquad k_{x,\text{inc}} = k_0 \sin\theta_{\text{inc}}, \qquad k_{z,\text{inc}} = k_0 \cos\theta_{\text{inc}},$$

$$k_{z,m} = \sqrt{k_m^2 - k_{x,\text{inc}}^2}, \qquad k_{z,m}^{\text{hyp}} = \sqrt{\epsilon_t \left(k_m^2 - \frac{k_{x,\text{inc}}^2}{\epsilon_z} \right)},$$

and the angle associated with each harmonic in regions I and III is

$$\theta_m = \arcsin\left(\frac{k_{x,\text{inc}}}{k_m} \right), \qquad |\theta_m| < \tfrac{\pi}{2}.$$

For evanescent orders $k_{z,m}$ is purely imaginary with $\text{Im}[k_{z,m}] < 0$, and $\theta_0 = \theta_{\text{inc}}$.

From the region-I expression it is clear that the incident field is

$$\mathbf{H}_{\text{inc}}(x, z; t) = e^{-jk_{x,\text{inc}}x} e^{-jk_{z,\text{inc}}z} e^{j\omega_0 t}\, \hat{\mathbf{y}}.$$

Using the TM relation

$$\mathbf{E} = \eta\, \mathbf{H} \times \frac{\mathbf{k}}{|\mathbf{k}|},$$

we obtain the incident electric field

$$\mathbf{E}_{\text{inc}}(x, z; t) = \eta\, e^{-jk_{x,\text{inc}}x} e^{-jk_{z,\text{inc}}z} e^{j\omega_0 t} \Big(\cos\theta_{\text{inc}}\, \hat{\mathbf{x}} - \sin\theta_{\text{inc}}\, \hat{\mathbf{z}} \Big).$$

For each harmonic in free space (regions I and III), we write

$$\mathbf{H}_m = H_m \hat{\mathbf{y}}, \qquad \mathbf{k}_m = k_{x,\text{inc}} \hat{\mathbf{x}} + k_{z,m} \hat{\mathbf{z}} = k_m (\sin\theta_m \hat{\mathbf{x}} + \cos\theta_m \hat{\mathbf{z}}),$$

which leads to

$$k_{z,m} > 0: \quad \mathbf{E}_m = -\eta H_m \sin\theta_m\, \hat{\mathbf{z}} + \eta H_m \cos\theta_m\, \hat{\mathbf{x}},$$

$$k_{z,m} < 0: \quad \mathbf{E}_m = -\eta H_m \sin\theta_m\, \hat{\mathbf{z}} - \eta H_m \cos\theta_m\, \hat{\mathbf{x}}.$$

Inside the hyperbolic medium, using Maxwell's equations with $\underline{\underline{\epsilon_r}} = \text{diag}(\epsilon_t, \epsilon_t, \epsilon_z)$ gives, for each

ω_m,

$$E^{\mathrm{II}}_{x,m} = \eta \, \frac{k^{\mathrm{hyp}}_{z,m}}{k_m \epsilon_t} H^{\mathrm{II}}_m, \qquad E^{\mathrm{II}}_{z,m} = -\eta \, \frac{k_{x,\mathrm{inc}}}{k_m \epsilon_z} H^{\mathrm{II}}_m,$$

which directly leads to the series expressions in your original formulation.

Multiharmonic vector and operator notation. We now collect all harmonic amplitudes into column vectors (single underline) and introduce matrix operators (double underline) in the harmonic space:

$$\underline{r} \triangleq (\ldots, r_{-2}, r_{-1}, r_0, r_{+1}, r_{+2}, \ldots)^{\mathsf{T}}, \quad \underline{a},\ \underline{b},\ \underline{t} \text{ defined similarly,}$$

$$\underline{1_0} \triangleq (\ldots, 0, 0, 1, 0, 0, \ldots)^{\mathsf{T}},$$

$$\underline{\underline{k}} = \mathrm{diag}(\ldots, k_{-2}, k_{-1}, k_0, k_{+1}, k_{+2}, \ldots), \quad \underline{\underline{k_z}} = \mathrm{diag}(\ldots, k_{z,-2}, k_{z,-1}, k_{z,0}, k_{z,+1}, k_{z,+2}, \ldots),$$

$$\underline{\underline{k_z^{\mathrm{hyp}}}} = \mathrm{diag}(\ldots, k^{\mathrm{hyp}}_{z,-2}, k^{\mathrm{hyp}}_{z,-1}, k^{\mathrm{hyp}}_{z,0}, k^{\mathrm{hyp}}_{z,+1}, k^{\mathrm{hyp}}_{z,+2}, \ldots),$$

and

$$\underline{\underline{\theta}} = \mathrm{diag}(\ldots, \theta_{-2}, \theta_{-1}, \theta_0, \theta_{+1}, \theta_{+2}, \ldots).$$

Based on these, we define the diagonal "ratio" operator

$$\underline{\underline{k_z^{\mathrm{hyp}}}}\, \underline{\underline{k}}^{-1} = \mathrm{diag}\Big(\ldots, \frac{k^{\mathrm{hyp}}_{z,-2}}{k_{-2}}, \frac{k^{\mathrm{hyp}}_{z,-1}}{k_{-1}}, \frac{k^{\mathrm{hyp}}_{z,0}}{k_0}, \ldots\Big),$$

and the exponential and cosine operators

$$e^{\pm j \underline{\underline{k_z}} z} \triangleq \mathrm{diag}(\ldots, e^{\pm j k_{z,-2} z}, \ldots, e^{\pm j k_{z,+2} z}, \ldots),$$

$$e^{\pm j \underline{\underline{k_z^{\mathrm{hyp}}}} z} \triangleq \mathrm{diag}(\ldots, e^{\pm j k^{\mathrm{hyp}}_{z,-2} z}, \ldots, e^{\pm j k^{\mathrm{hyp}}_{z,+2} z}, \ldots),$$

$$\cos(\underline{\underline{\theta}}) \triangleq \mathrm{diag}(\ldots, \cos\theta_{-2}, \cos\theta_{-1}, \cos\theta_0, \cos\theta_{+1}, \cos\theta_{+2}, \ldots).$$

In this notation, the multiharmonic magnetic fields in the three regions can be written concisely

as

$$\underline{\mathbf{H}}^{\mathrm{I}}(x,z) = e^{-jk_{x,\mathrm{inc}}x}\Big[e^{-jk_{z,\mathrm{inc}}z}\,\underline{1_0} + e^{+j\underline{\underline{k_z}}z}\,\underline{r}\Big]\hat{\mathbf{y}}, \tag{S13}$$

$$\underline{\mathbf{H}}^{\mathrm{II}}(x,z) = e^{-jk_{x,\mathrm{inc}}x}\Big[e^{-j\underline{\underline{k_z^{\mathrm{hyp}}}}z}\,\underline{a} + e^{+j\underline{\underline{k_z^{\mathrm{hyp}}}}z}\,\underline{b}\Big]\hat{\mathbf{y}}, \tag{S14}$$

$$\underline{\mathbf{H}}^{\mathrm{III}}(x,z) = e^{-jk_{x,\mathrm{inc}}x}e^{-j\underline{\underline{k_z}}z}\,\underline{t}\,\hat{\mathbf{y}}. \tag{S15}$$

Similarly, the tangential electric field components in each region follow from the relations above. For the x-components we obtain

$$\underline{\mathbf{E}}_x^{\mathrm{I}}(x,z) = \eta\, e^{-jk_{x,\mathrm{inc}}x}\Big[\cos\theta_{\mathrm{inc}}\, e^{-jk_{z,\mathrm{inc}}z}\,\underline{1_0} - \cos(\underline{\underline{\theta}})\, e^{+j\underline{\underline{k_z}}z}\,\underline{r}\Big]\hat{\mathbf{x}},$$

$$\underline{\mathbf{E}}_x^{\mathrm{II}}(x,z) = \eta\, e^{-jk_{x,\mathrm{inc}}x}\,\frac{1}{\epsilon_t}\,\underline{\underline{k_z^{\mathrm{hyp}}}}\,\underline{\underline{k}}^{-1}\Big[e^{-j\underline{\underline{k_z^{\mathrm{hyp}}}}z}\,\underline{a} - e^{+j\underline{\underline{k_z^{\mathrm{hyp}}}}z}\,\underline{b}\Big]\hat{\mathbf{x}},$$

$$\underline{\mathbf{E}}_x^{\mathrm{III}}(x,z) = \eta\, e^{-jk_{x,\mathrm{inc}}x}\,\cos(\underline{\underline{\theta}})\, e^{-j\underline{\underline{k_z}}z}\,\underline{t}\,\hat{\mathbf{x}}.$$

(The corresponding z-components follow analogously from the hyperbolic constitutive relations and are not written explicitly here, since only the tangential components enter the sheet boundary conditions.)

Time-modulated conductive sheets and boundary conditions. The two time-modulated conductive sheets at $z = 0$ and $z = L$ are described by

$$\mathbf{J}_s(t) = \sigma(t)\,\mathbf{E}_t(t),$$

with T-periodic surface conductance

$$\sigma(t) = \sum_n \sigma_n e^{jn\Omega t}.$$

In harmonic space this corresponds to a Toeplitz matrix

$$\underline{\underline{\sigma}} = \Big[\sigma_{p-q}\Big]_{p,q\in\mathbb{Z}},$$

such that $(\underline{\underline{\sigma}}\,\underline{\mathbf{E}})_m = \sum_n \sigma_{m-n}\mathbf{E}_n$. We denote by $\underline{\underline{\sigma}}^0$ and $\underline{\underline{\sigma}}^L$ the Toeplitz matrices associated with the sheets at $z = 0$ and $z = L$, respectively.

The boundary conditions follow from continuity of the tangential electric field E_x and from

$$\hat{\mathbf{z}} \times \left(\mathbf{H}^{\mathrm{up}} - \mathbf{H}^{\mathrm{down}}\right) = \mathbf{J}_s(t) = \sigma(t)\,\mathbf{E}_t(t),$$

applied at $z = 0$ and $z = L$ in the multiharmonic representation. Using the compact forms above, we obtain the following matrix system for the unknown vectors $\underline{r}$, $\underline{a}$, $\underline{b}$, and $\underline{t}$:

$$\begin{aligned}
\cos(\underline{\underline{\theta}})\left(\underline{1_0} - \underline{r}\right) &= \frac{1}{\epsilon_t}\,\underline{\underline{k_z^{\mathrm{hyp}}}}\,\underline{\underline{k}}^{-1}\left(\underline{a} - \underline{b}\right), \\
\frac{1}{\epsilon_t}\,\underline{\underline{k_z^{\mathrm{hyp}}}}\,\underline{\underline{k}}^{-1}\left[e^{-j\underline{\underline{k_z^{\mathrm{hyp}}}}L}\,\underline{a} - e^{+j\underline{\underline{k_z^{\mathrm{hyp}}}}L}\,\underline{b}\right] &= \cos(\underline{\underline{\theta}})\,e^{-j\underline{\underline{k_z}}L}\,\underline{t}, \\
\underline{1_0} + \underline{r} - \underline{a} - \underline{b} &= \eta\,\underline{\underline{\sigma}}^0\,\cos(\underline{\underline{\theta}})\left(\underline{1_0} - \underline{r}\right), \\
e^{-j\underline{\underline{k_z^{\mathrm{hyp}}}}L}\,\underline{a} + e^{+j\underline{\underline{k_z^{\mathrm{hyp}}}}L}\,\underline{b} - e^{-j\underline{\underline{k_z}}L}\,\underline{t} &= \eta\,\underline{\underline{\sigma}}^L\,e^{-j\underline{\underline{k_z}}L}\,\cos(\underline{\underline{\theta}})\,\underline{t}.
\end{aligned}$$

By solving this linear system for $\underline{r}$, $\underline{a}$, $\underline{b}$, and $\underline{t}$, we obtain all reflection and transmission harmonics of the microwave configuration. In this notation, single underlines denote multiharmonic column vectors, and double underlines denote matrix operators (including diagonal and Toeplitz matrices) acting in the harmonic space.

Performance analysis of the microwave device in more detail

Figure S8 illustrates the multiharmonic surface currents generated on the two time-modulated conductive sheets. Since each sheet is described through the Fourier expansion

$$\sigma(t) = \sum_n \sigma_n e^{jn\Omega t},$$

the induced surface current,

$$\mathbf{J}_s(t) = \sigma(t)\mathbf{E}(t),$$

naturally inherits a multiharmonic structure. Writing the fields in Floquet form,

$$\mathbf{E}(t) = \sum_m \mathbf{E}_m e^{jm\Omega t}, \qquad \mathbf{J}_s(t) = \sum_m \mathbf{J}_{s,m} e^{jm\Omega t},$$

gives the harmonic-mixing relation

$$\mathbf{J}_{s,m} = \sum_n \sigma_n \, \mathbf{E}_{m-n},$$

which follows from the multiply–convolve property of Fourier transforms. Figure S8 directly visualizes this mechanism: the two sheets generate distinct multiharmonic current patterns under forward versus backward excitation, and the redistribution among neighboring sidebands reflects the strength and phase of the temporal modulation.

Figures S9 and S10 show how the incident power ($P_{\text{inc}} = 1$ W/m) is distributed among the Floquet harmonics inside the microwave structure. Similar to the optical device, the temporal modulation continuously injects energy into sidebands, while the hyperbolic slab selectively enhances or suppresses different harmonics depending on their longitudinal wavenumbers. Although the forward/backward contrast is somewhat milder than in the optical configuration, the internal harmonic power flow remains direction-dependent, further confirming the nonreciprocal behavior of the structure.

Supplementary Video 2 shows the time-domain evolution of the magnetic-field, $H_y(x, z, t)$, at the operating frequency ω_0, again corresponding to the zeroth-order Floquet harmonic, for forward and backward excitation of the microwave device. As in the optical case, the video uses a nonlinear μ-law colormap with $\mu = 10$ to compress the dynamic range and make the directional difference in transmitted amplitude visually prominent. Although the forward/backward contrast is milder than in the optical configuration, the nonlinear scaling allows the isolation to be easily perceived. This dynamic visualization complements Figs. S9 and S10 by showing how the fundamental harmonic evolves in time in response to the temporally modulated conductive sheets, thereby clarifying the origin of the device's nonreciprocal behavior.

Figure S11 presents the spatial field profiles for forward and backward illumination. The

transmitted beam clearly refracts toward the negative side of the normal, demonstrating negative refraction in the microwave implementation. In addition, the field distribution inside the slab differs between the two excitation directions due to the distinct modal interference pathways generated by the temporally modulated sheets.

Finally, Figure S12 examines how device performance varies with the modulation-phase difference between the two time-varying sheets. The insertion loss, previously defined in the corresponding optical section, exhibits its minimum near the quadrature point $\phi_2 - \phi_1 = \pi/2$, consistent with the theoretical condition predicted by temporal coupled-mode theory.[S1] Unlike the optical device, where finite-thickness and multimode coupling slightly shift the optimum phase, the microwave configuration reaches its best performance close to the ideal $\pi/2$ value.

Power exchange calculations in the presence of time-varying conductive sheets

Since the microwave device contains no time-varying bulk dielectric regions, we begin directly from the standard Poynting theorem,

$$\nabla \cdot \mathbf{S} + \frac{\partial}{\partial t}\Big(\tfrac{1}{2}\,\mathbf{E}\cdot\mathbf{D} + \tfrac{1}{2}\,\mathbf{H}\cdot\mathbf{B}\Big) = -\,\mathbf{E}\cdot\mathbf{J}_{\mathrm{free}}, \tag{S16}$$

where the tangential sheet current density is included in $\mathbf{J}_{\mathrm{free}}$. For a time-modulated conductive sheet with instantaneous conductivity $\sigma(t)$, the instantaneous Ohmic power density is

$$p_\sigma(t) = \mathbf{E}(t)\cdot\mathbf{J}_{\mathrm{free}}(t) = \sigma(t)\,|\mathbf{E}(t)|^2. \tag{S17}$$

To determine how the modulation modifies power absorption, we compute the time average of $p_\sigma(t)$ over one modulation period $T = 2\pi/\Omega$ using real-field (cosine) Fourier expansions with peak phasors and the convention $e^{+j\omega t}$.

Real-field Fourier representation and average dissipation

The physical electric field is represented as the real part of a multiharmonic expansion,

$$\mathbf{E}(t) = \Re\left\{ \sum_{m\in\mathbb{Z}} \mathbf{E}_m e^{j(\omega_0+m\Omega)t} \right\} = \tfrac{1}{2} \sum_{m} \left(\mathbf{E}_m e^{j(\omega_0+m\Omega)t} + \mathbf{E}_m^* e^{-j(\omega_0+m\Omega)t} \right), \tag{S18}$$

where $\mathbf{E}_m$ denote the peak complex amplitudes of the Floquet harmonics. The squared magnitude $|\mathbf{E}(t)|^2$ inherits an Ω-periodic structure; after discarding the rapidly oscillating $2\omega_0$ components, it may be written as

$$|\mathbf{E}(t)|^2 = \sum_{k\in\mathbb{Z}} \widetilde{C}_k \, e^{jk\Omega t}, \qquad \widetilde{C}_k = \tfrac{1}{2} \sum_{n} \mathbf{E}_{n+k} \cdot \mathbf{E}_n^*, \qquad \widetilde{C}_{-k} = \widetilde{C}_k^*. \tag{S19}$$

The sheet conductivity is expanded similarly. For single-tone modulation,

$$\sigma(t) = \sigma_0 + \Delta\sigma \cos(\Omega t + \phi) = S_0 + S_{+1} e^{j\Omega t} + S_{-1} e^{-j\Omega t}, \qquad S_0 = \sigma_0, \quad S_{\pm 1} = \tfrac{\Delta\sigma}{2} e^{\pm j\phi}. \tag{S20}$$

Multiplying $\sigma(t)$ by $|\mathbf{E}(t)|^2$ yields another Ω-periodic series. The time average $\langle p_\sigma \rangle$ corresponds to the DC coefficient, which is formed by all pairs of Fourier indices whose exponents sum to zero:

$$\langle p_\sigma \rangle = S_0 \, \widetilde{C}_0 + S_{+1} \widetilde{C}_{-1} + S_{-1} \widetilde{C}_{+1} \tag{S21}$$

$$= \tfrac{1}{2} \, \sigma_0 \sum_{n} |\mathbf{E}_n|^2 + \tfrac{1}{2} \, \Delta\sigma \, \Re\left\{ e^{-j\phi} \sum_{n} \mathbf{E}_{n+1} \cdot \mathbf{E}_n^* \right\}. \tag{S22}$$

The first term is the familiar static Ohmic loss, while the second term describes the modulation-induced correction. Notably, this correction depends on the *real* part of the cross-correlation between adjacent sidebands, in contrast with the time-varying-permittivity case (optical device), where the exchange term depended on the imaginary part of the corresponding correlation.

Surface-current formulation of the power exchange

For a conductive sheet, the instantaneous surface power density is

$$p_s(t) = \mathbf{E}_t(t) \cdot \mathbf{J}_s(t), \tag{S23}$$

where $\mathbf{E}_t$ is the tangential electric field. Using real-field Floquet representations,

$$\mathbf{E}_t(t) = \Re\left\{\sum_m \mathbf{E}_m e^{j(\omega_0+m\Omega)t}\right\}, \qquad \mathbf{J}_s(t) = \Re\left\{\sum_m \mathbf{J}_m e^{j(\omega_0+m\Omega)t}\right\}, \tag{S24}$$

the product $\mathbf{E}_t \cdot \mathbf{J}_s$ contains many oscillatory terms, but only those with zero net modulation frequency contribute to the time average. This yields the compact relation

$$\langle p_s \rangle = \tfrac{1}{2}\,\Re\left\{\sum_{n\in\mathbb{Z}} \mathbf{E}_n \cdot \mathbf{J}_n^*\right\}. \tag{S25}$$

The factor $\frac{1}{2}$ appears because the above uses peak phasors and real-part reconstructions.

Figure S13 illustrates these mechanisms for the two time-modulated conductive sheets. In Fig. S13(a), the decomposition into the static Ohmic term and the modulation-induced contribution clearly shows how harmonic mixing among adjacent Floquet components alters the net dissipation on each sheet. Finally, Fig. S13(b) compares forward and backward illumination. It can be observed that, for forward illumination, the conductive sheets exhibit lower absorption (0.7 dB less), leading to higher transmission, which results in an isolation of 11.4 dB.

References

(S1) Chegnizadeh, M.; Memarian, M.; Mehrany, K. Non-reciprocity using quadrature-phase time-varying slab resonators. *Journal of the Optical Society of America B* **2020**, *37*, 88–97.

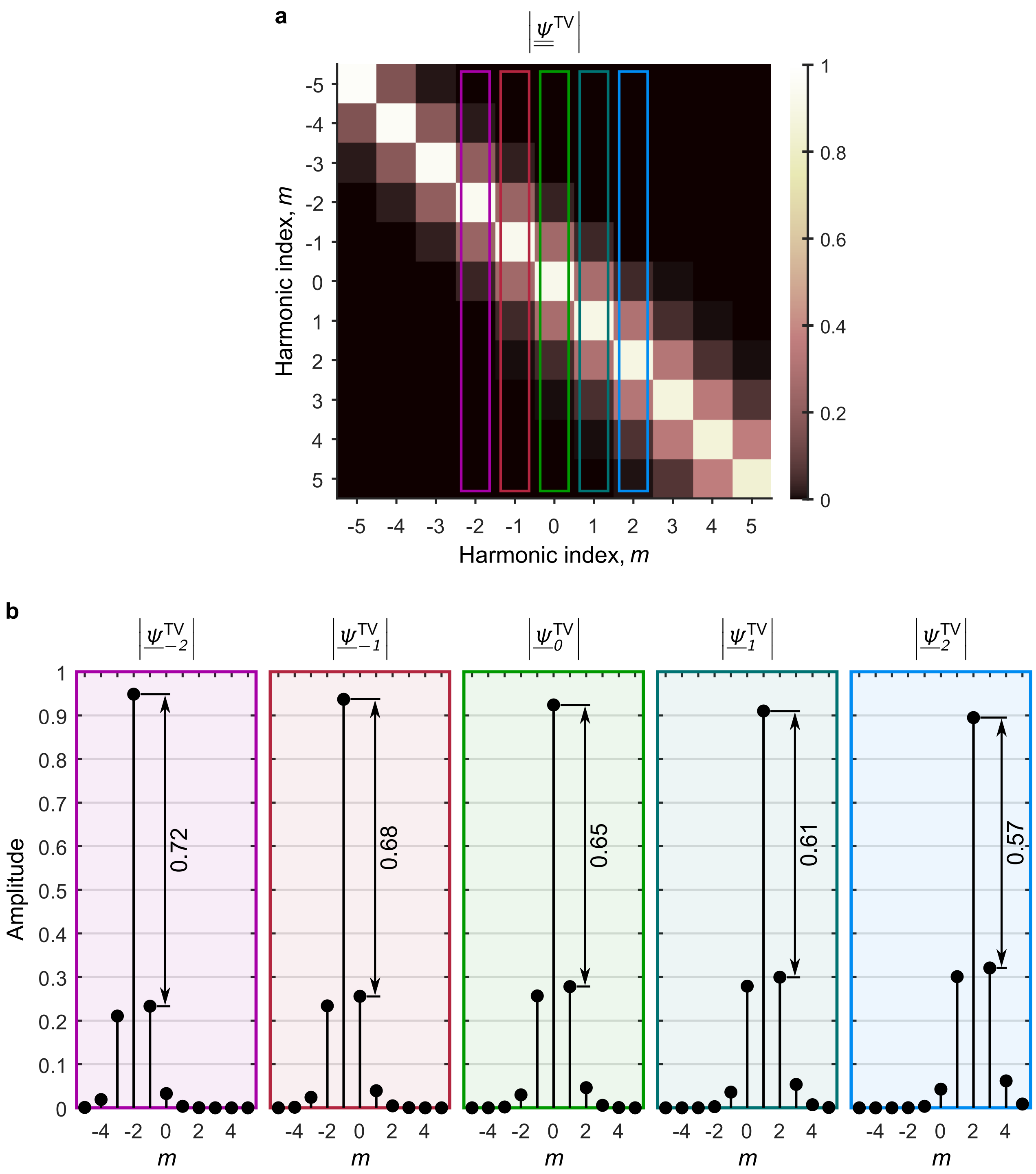

Figure S1: Multiharmonic eigenmodes of the two time-varying (TV) slabs. (a) Amplitude map of the eigenfunction matrices $\underline{\underline{\Psi}}^{\mathrm{TV1}}$ and $\underline{\underline{\Psi}}^{\mathrm{TV2}}$, whose columns correspond to the dominant multiharmonic modes supported by each temporally modulated slab. The colored rectangles highlight the five principal modes, $\underline{\Psi}_{-2}^{\mathrm{TV}}$, $\underline{\Psi}_{-1}^{\mathrm{TV}}$, $\underline{\Psi}_{0}^{\mathrm{TV}}$, $\underline{\Psi}_{+1}^{\mathrm{TV}}$, and $\underline{\Psi}_{+2}^{\mathrm{TV}}$, each of which comprises a mixture of harmonics indexed by m. (b) Stem plots of the harmonic amplitudes for the five modes identified in (a). For each multiharmonic mode, the relative weights of the primary harmonic and its side harmonics are shown, illustrating how the temporal modulation redistributes energy across adjacent frequency components. Each stem-plot panel is color-matched to its corresponding column in (a).

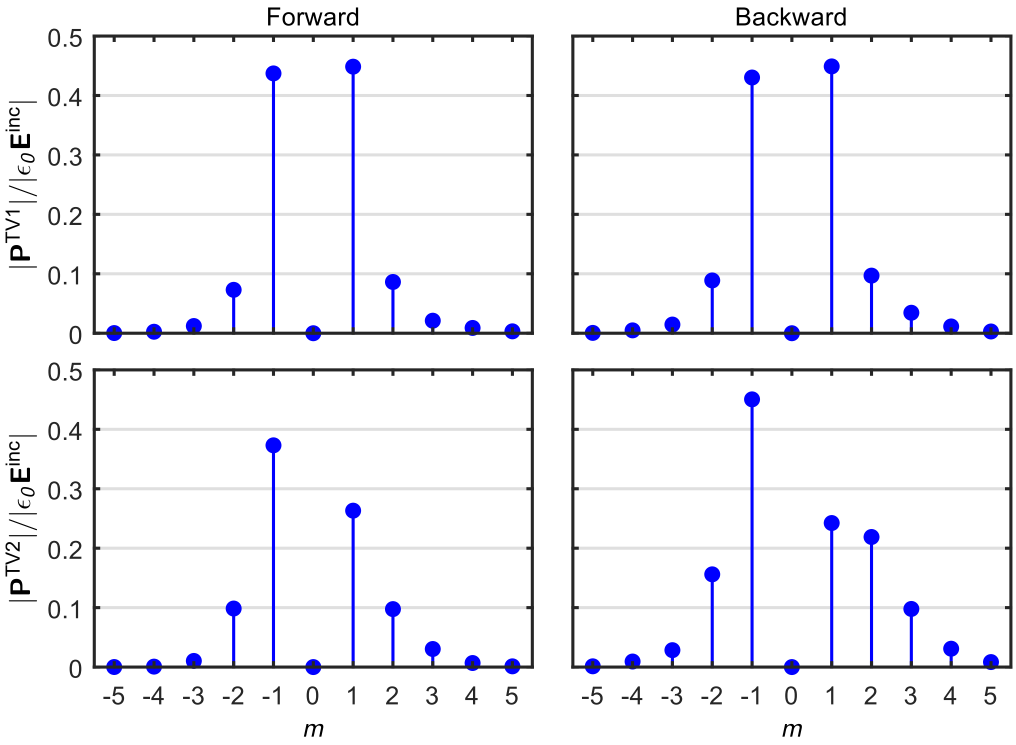

Figure S2: Harmonic polarization amplitudes induced in the two time-varying slabs. Polarization amplitudes of the generated Floquet harmonics in the two temporally modulated slabs, computed under forward and backward illumination. Each stem plot shows the magnitude of the volumetric polarization at harmonic index m, normalized to the incident flux density. The distributions highlight how temporal modulation excites distinct sets of harmonics in the forward and backward directions, reflecting the direction-dependent coupling that underlies the nonreciprocal response.

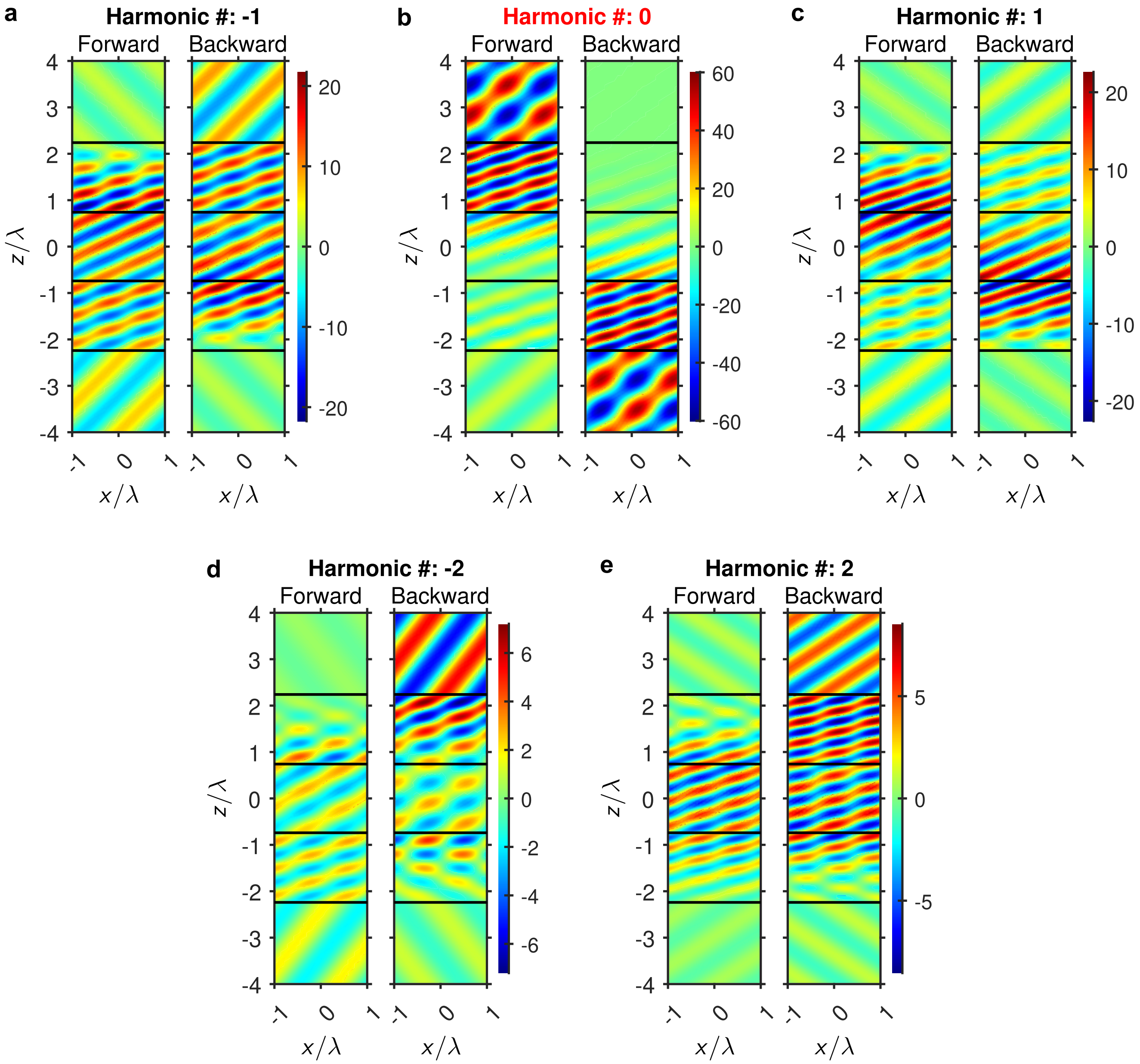

Figure S3: Magnetic-field distributions at the harmonic frequencies $\omega_m = \omega_0 + m\Omega$ for the optical device. Spatial distributions of the magnetic field component $H_y(x, z)$ for the dominant Floquet harmonics generated inside the temporally modulated dielectric slabs of the optical device, under both forward and backward illumination. Panels show the field maps at the harmonic frequencies: (a) $\omega_{-1} = \omega_0 - \Omega$, (b) ω_0, (c) $\omega_{+1} = \omega_0 + \Omega$, (d) $\omega_{-2} = \omega_0 - 2\Omega$, and (e) $\omega_{+2} = \omega_0 + 2\Omega$. For each harmonic, the left and right subpanels correspond to forward and backward incidence, respectively. All maps for the same harmonic index m share a common linear colormap scale, enabling direct comparison of forward/backward amplitudes and illustrating the direction-dependent harmonic content produced by the time-modulated slabs.

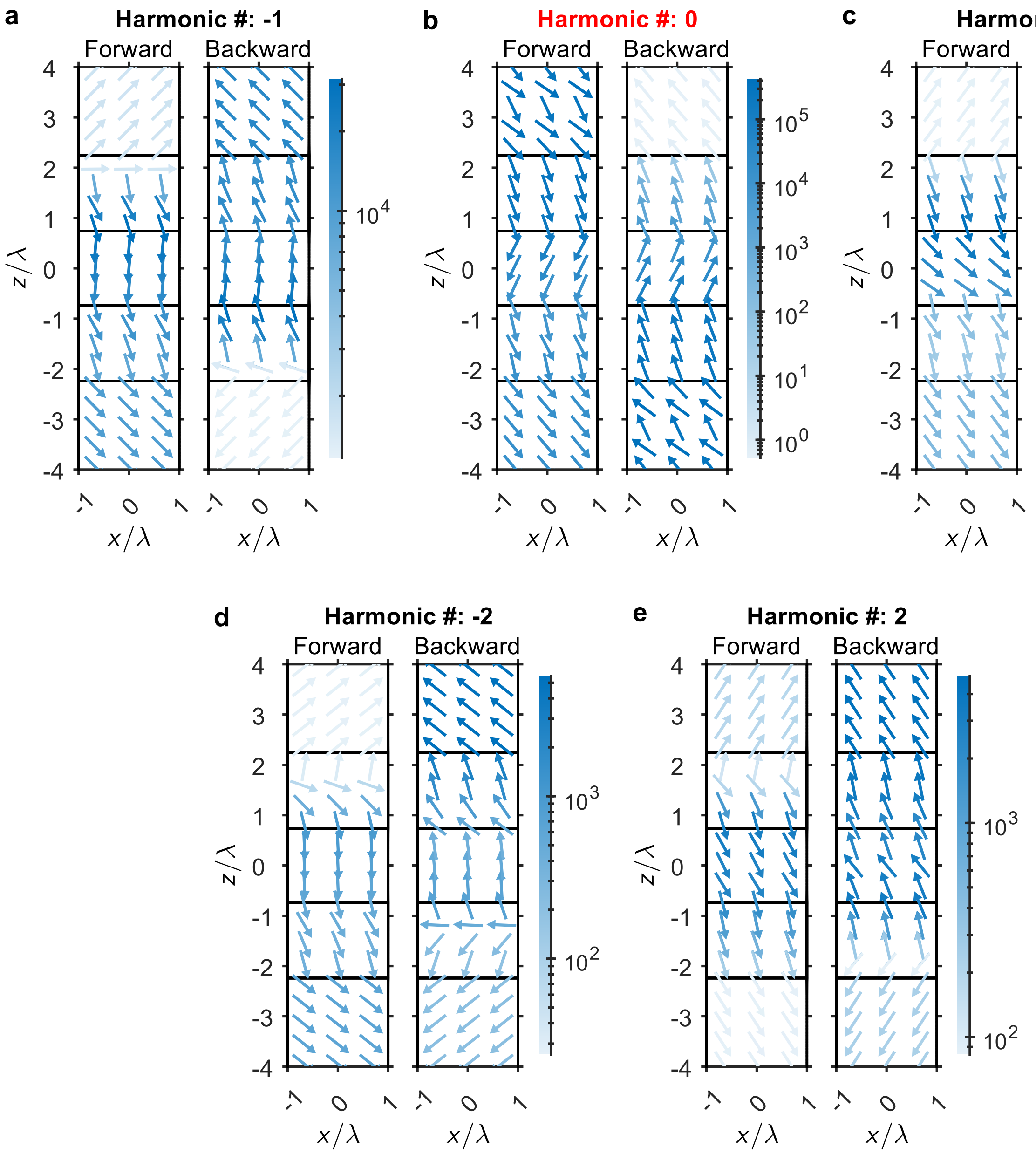

Figure S4: Power-flow distributions of individual Floquet harmonics in the optical device. Time-averaged Poynting vector fields for the principal harmonics generated in the temporally modulated dielectric slabs of the optical device, shown under forward and backward illumination. Panels correspond to (a) $m = -1$, (b) $m = 0$, (c) $m = +1$, (d) $m = -2$, and (e) $m = +2$, where each harmonic occurs at $\omega_m = \omega_0 + m\Omega$. For every harmonic, the left and right subpanels display the forward- and backward-incidence power-flow patterns, respectively. All panels use a common logarithmic colormap scale to highlight the broad dynamic range of power amplitudes and to reveal subtle directional differences in harmonic energy transport.

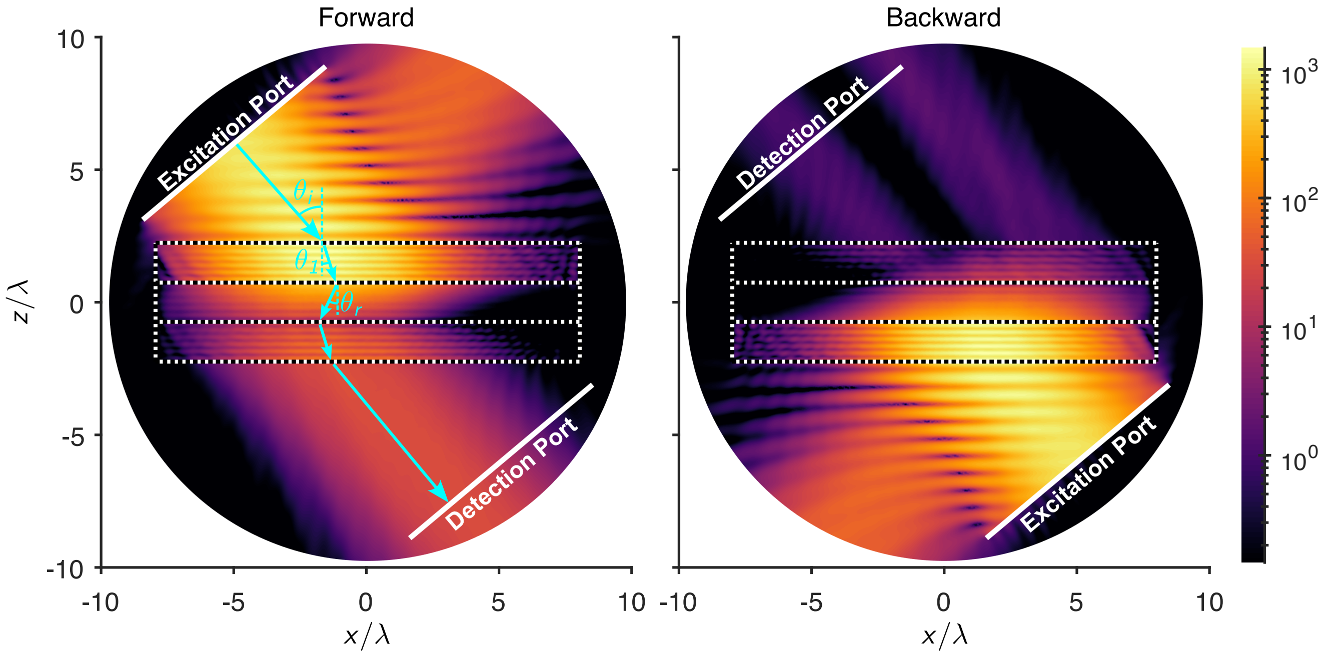

Figure S5: Gaussian-beam negative refraction through the optical device including the time-varying dielectric slabs. Magnetic-field intensity distributions $|\mathbf{H}|^2$ at the operating frequency ω_0 for forward and backward Gaussian-beam illumination at an incident angle of $\theta_i = 40°$. The dotted black–white lines denote the boundaries of the optical structure, in which the top and bottom regions are the temporally modulated dielectric slabs and the central region is the hyperbolic medium. Both surface plots use a common logarithmic color scale spanning a 40-dB dynamic range. Cyan arrows indicate the refraction trajectories, highlighting the negative-refraction behavior inside the hyperbolic core. From the simulated field patterns, the refraction angle at the air–modulated-slab interface is $\theta_1 = 18.7°$, and the refraction angle at the modulated-slab–hyperbolic interface is $\theta_r = -26.7°$, confirming the backward-wave propagation intrinsic to the hyperbolic region.

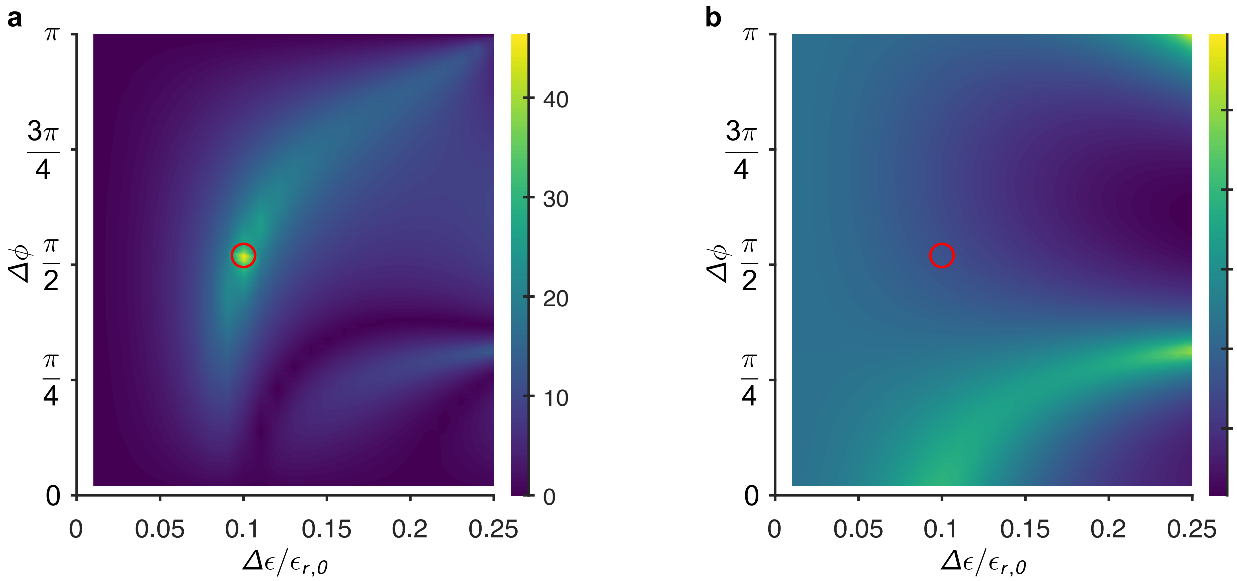

Figure S6: Influence of modulation parameters on the performance of the optical device. (a) Isolation of the optical device as a function of the modulation depth $\Delta\epsilon/\epsilon_{r,0}$ and the phase difference $\Delta\phi = \phi_2 - \phi_1$ between the two temporally modulated dielectric slabs, shown on a dB scale. (b) Forward-mode insertion loss versus the same modulation parameters, also presented in dB. In both panels, the red circle indicates the selected operating point of the device and its corresponding performance, illustrating the trade-off between isolation and insertion loss across the modulation-parameter space.

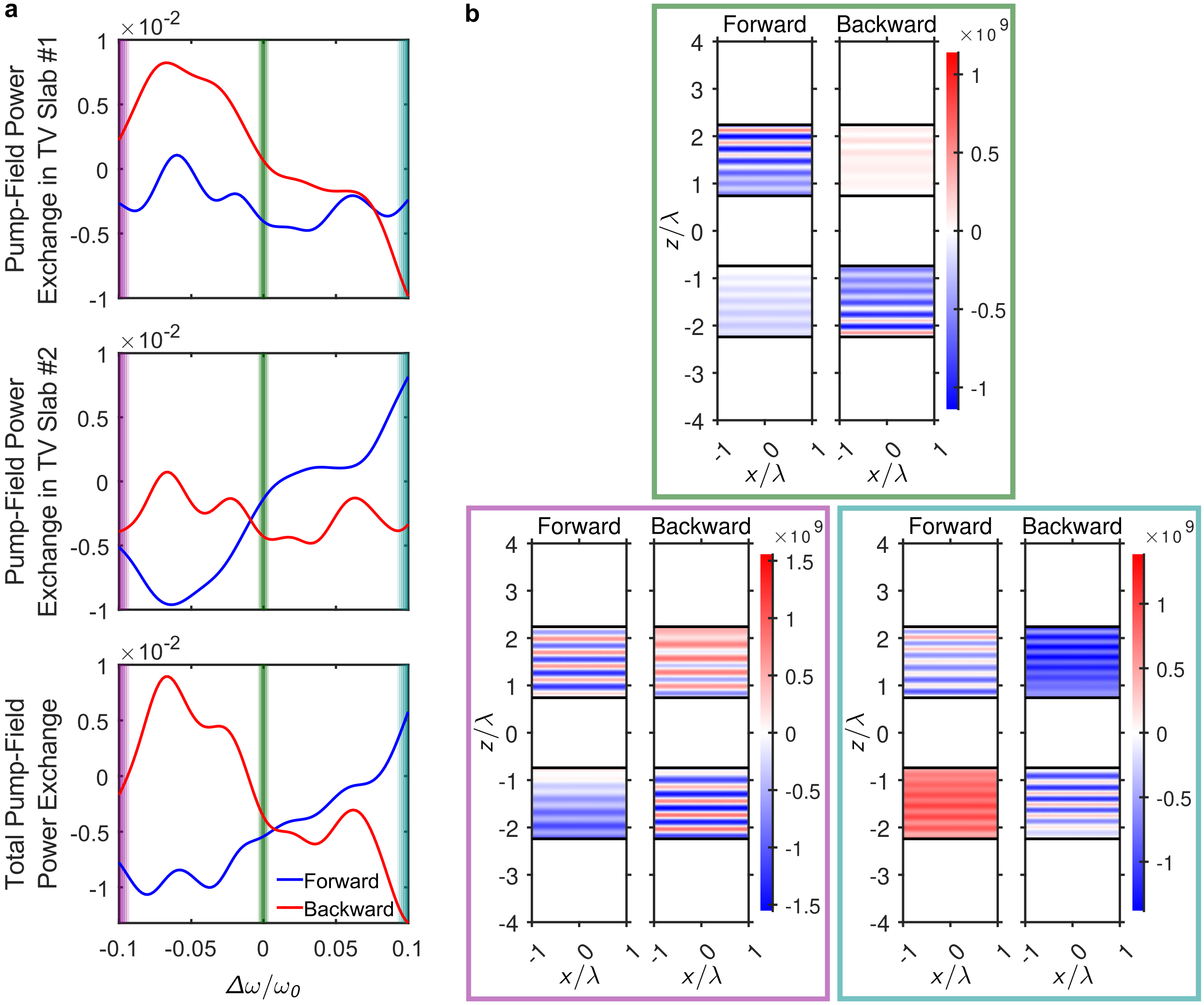

Figure S7: Pump-field power exchange within the temporally modulated slabs of the optical device. (a) Pump-field power exchange in the two time-varying dielectric slabs (TV Slab #1 and TV Slab #2) as a function of the normalized detuning $\Delta\omega/\omega_0$, shown for both forward and backward illumination. The bottom panel displays the total pump-field power exchange obtained by summing the contributions from the two slabs. Shaded vertical regions highlight the three representative detuning values used in panel (b). (b) Spatial distributions of the pump-field power exchange at detuning values of -10%, 0%, and $+10\%$, again under forward and backward incidence. Each distribution is enclosed within a colored frame matching the shaded detuning regions in panel (a). These profiles illustrate how the temporal modulation redistributes pump-field energy differently for the two incidence directions, and how this redistribution varies with detuning.

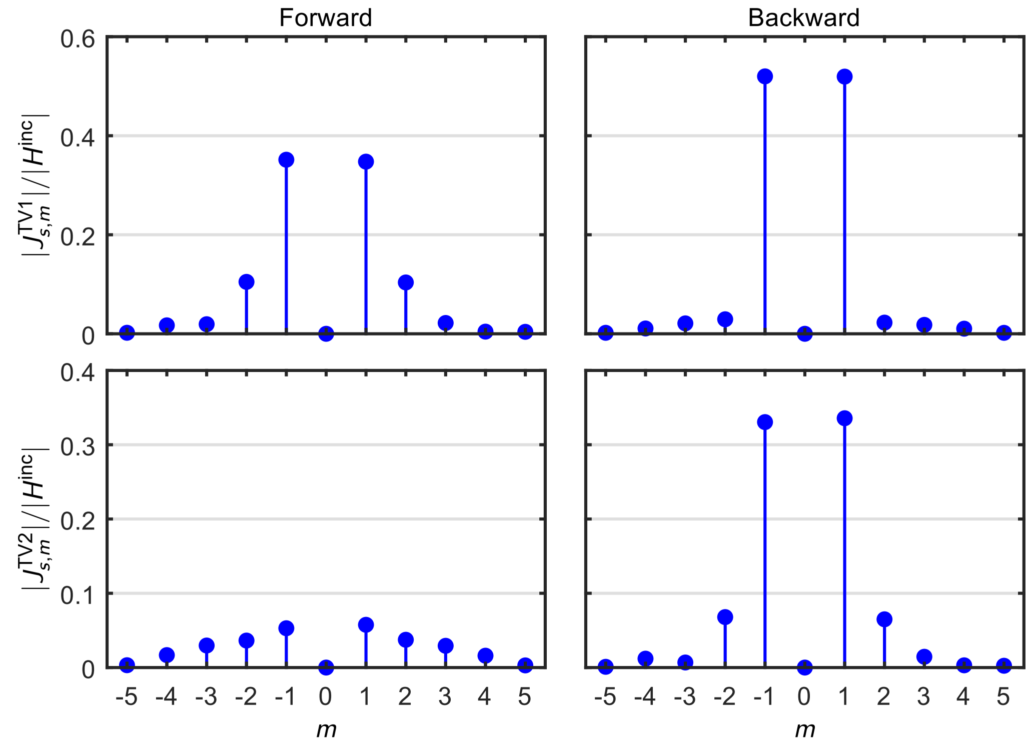

Figure S8: Harmonic surface-current amplitudes generated on the time-modulated sheets of the microwave device. Stem plots of the magnitudes of the multiharmonic surface currents $|J_{s,m}^{\mathrm{TV1}}|/|H^{\mathrm{inc}}|$ and $|J_{s,m}^{\mathrm{TV2}}|/|H^{\mathrm{inc}}|$ induced on the two temporally modulated conductive sheets under forward and backward illumination. Each panel reports the amplitude of the surface current associated with harmonic index m, normalized to the incident magnetic-field amplitude. These distributions illustrate how temporal modulation of the sheet conductance generates direction-dependent sets of harmonic surface currents, providing the mechanism for nonreciprocal functionality in the microwave device.

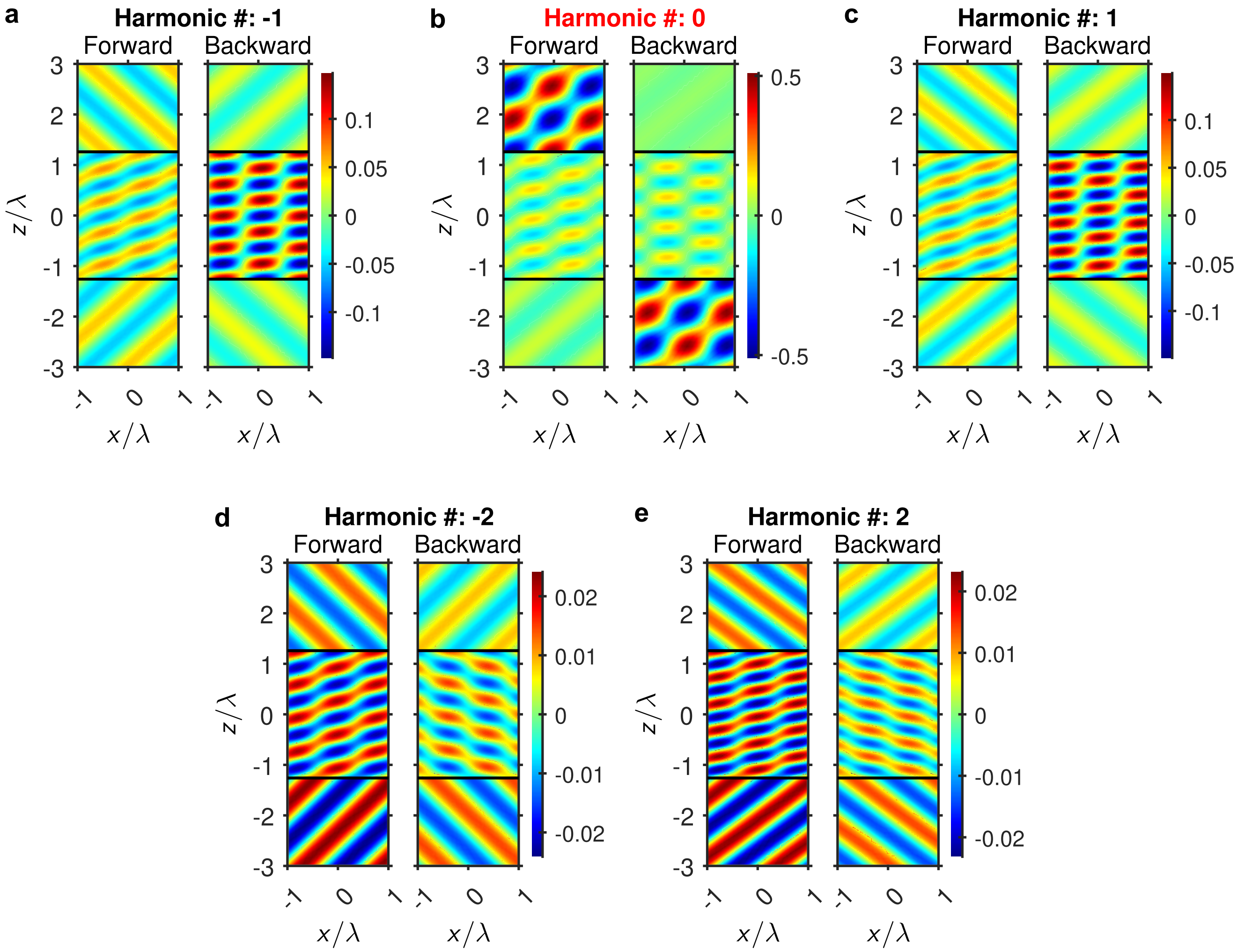

Figure S9: Spatial magnetic-field profiles of Floquet harmonics generated by the time-modulated sheets in the microwave device. Spatial distributions of the magnetic field $H_y(x, z)$ associated with the Floquet harmonics produced when the two conductive sheets undergo sinusoidal modulation of their surface conductance. The harmonics displayed are (a) $m = -1$, (b) $m = 0$, (c) $m = +1$, (d) $m = -2$, and (e) $m = +2$, corresponding to frequencies $\omega_m = \omega_0 + m\Omega$. Each panel contains forward-illumination and backward-illumination results side by side, enabling a direct comparison of how the modulated sheets interact differently with waves incident from opposite directions. A consistent linear colormap is used for each harmonic order.

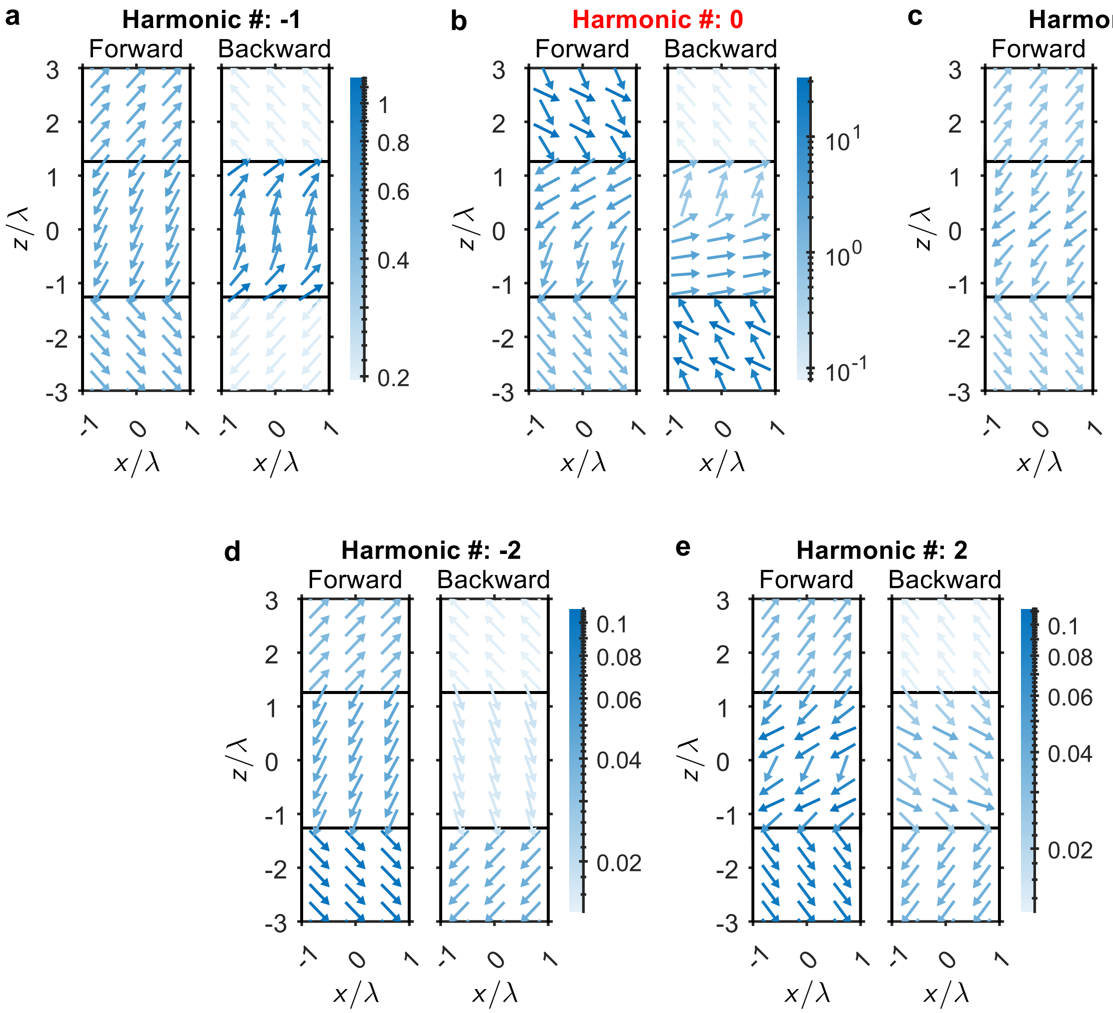

Figure S10: Harmonic power-flow patterns produced by the time-modulated conductive sheets in the microwave device. Time-averaged Poynting vector distributions corresponding to the Floquet harmonics generated by the modulated sheets, shown for forward and backward incident waves. Panels depict the energy-flow fields for (a) $m = -1$, (b) $m = 0$, (c) $m = +1$, (d) $m = -2$, and (e) $m = +2$, with each harmonic occurring at $\omega_m = \omega_0 + m\Omega$. A common logarithmic color-scale is employed for each harmonic to clearly illustrate variations in power magnitude over a broad dynamic range. These asymmetric power-flow characteristics reveal the mechanism by which temporally varying sheet conductances induce nonreciprocal energy transport through the microwave structure.

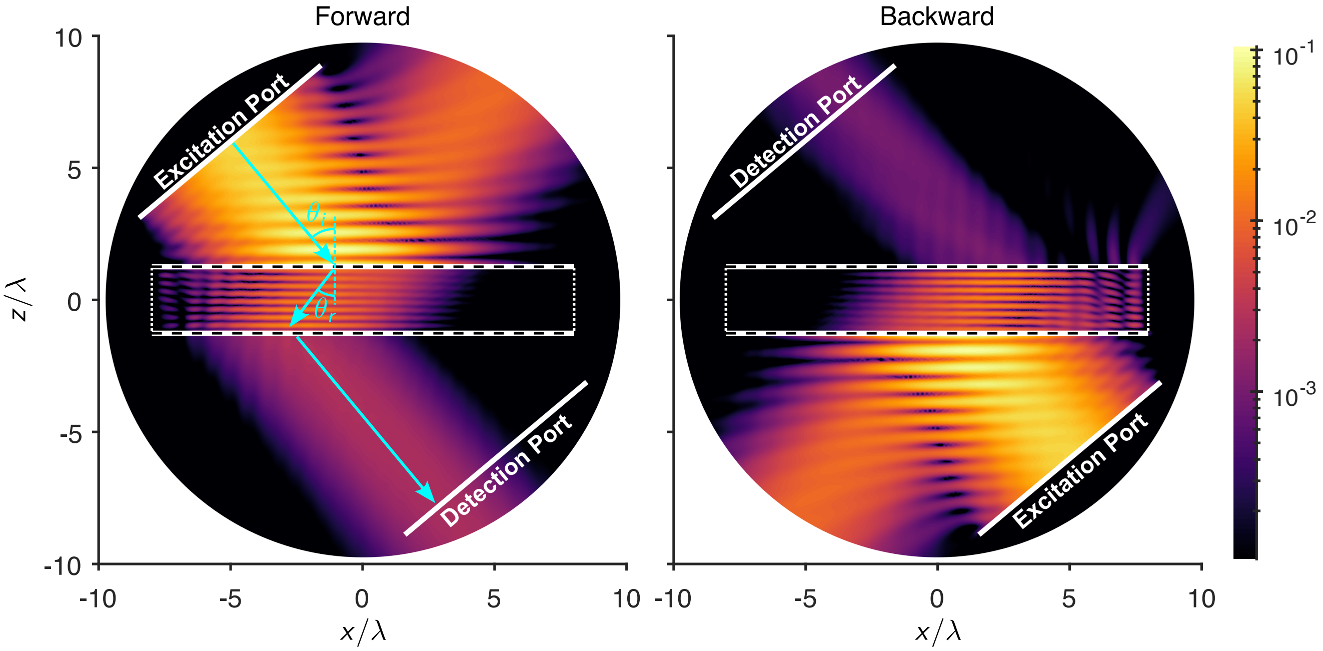

Figure S11: Gaussian-beam negative refraction through the microwave device with time-modulated conductive sheets. Magnetic-field intensity distributions $|\mathbf{H}|^2$ at the operating frequency ω_0 for Gaussian-beam illumination incident at $\theta_i = 40°$ in the forward and backward directions. The thick dashed black-white lines mark the locations of the two temporally modulated conductive sheets enclosing the hyperbolic metamaterial slab (wire medium). Both field maps use a common logarithmic color scale spanning a 30-dB dynamic range. The refraction trajectory under forward incidence is denoted by cyan arrows, illustrating negative refraction within the hyperbolic layer. The refraction angle at the interface between air and the hyperbolic medium (coincident with the modulated sheets) is $\theta_r = -37.4°$. The clear difference between forward and backward propagation reflects the asymmetric beam transmission induced by the temporal modulation of the conductive sheets.

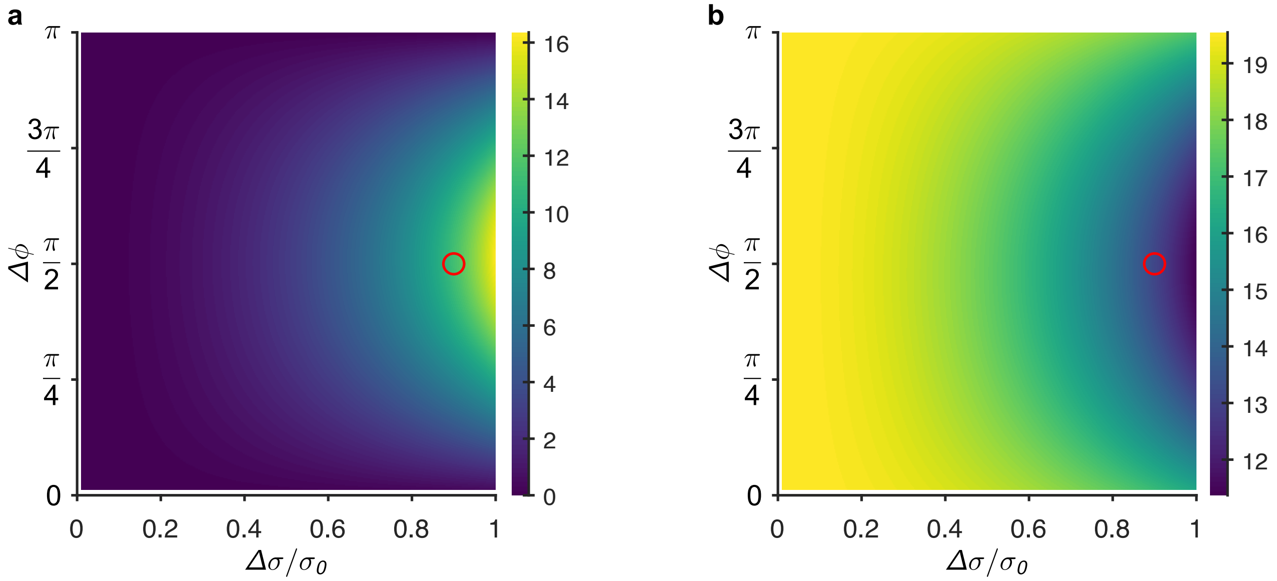

Figure S12: Dependence of microwave-device performance on conductance modulation depth and phase offset. (a) Isolation of the microwave device as a function of the normalized conductance modulation depth $\Delta\sigma/\sigma_0$ and the phase difference $\Delta\phi = \phi_2 - \phi_1$ between the two time-modulated conductive sheets. (b) Forward-mode insertion loss mapped over the same parameter space. In both panels, the red circle marks the chosen operating condition and the corresponding device performance. The color scales represent values in decibels.

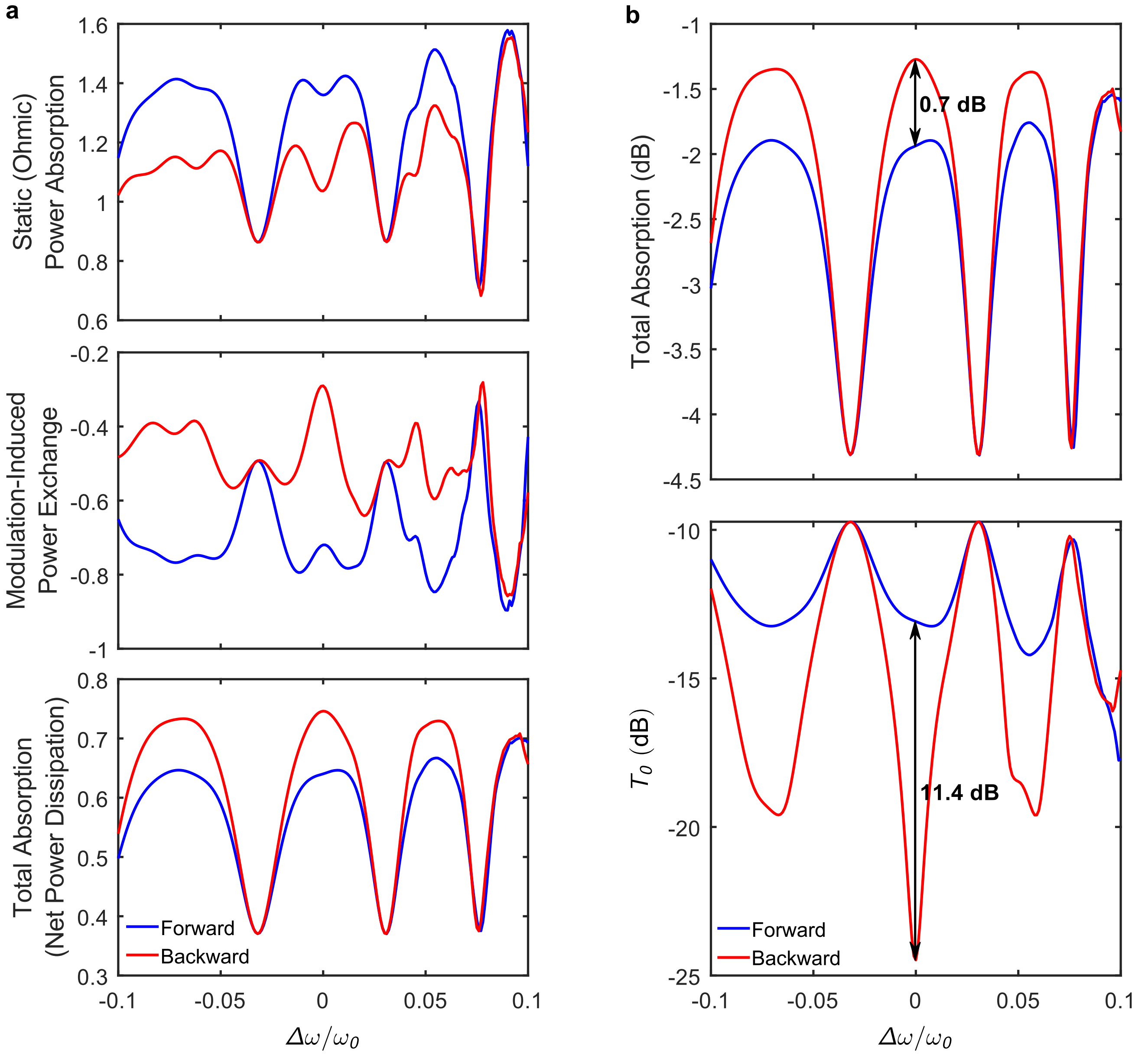

Figure S13: Power dissipation and pump-driven power exchange in the time-modulated conductive sheets of the microwave device. (a) Contributions to power dissipation within the two temporally modulated conductive sheets as functions of the normalized frequency detuning $\Delta\omega/\omega_0$. The top panel shows the static (DC) ohmic absorption originating from the baseline sheet conductivity σ_0. The middle panel displays the modulation-induced power exchange associated with sideband coupling due to the time-varying conductance; this contribution may take positive or negative values. The bottom panel reports the total net absorbed power, combining both mechanisms. All power quantities are given in absolute values and normalized to the incident plane-wave power, and both forward and backward incidences are shown. (b) Total absorption (upper panel, in dB) and zeroth-order transmission T_0 (lower panel, in dB) as functions of normalized frequency detuning for forward and backward illumination.
